# Variant cell cycles regulated by Notch signaling control cell size and ensure a functional blood-brain barrier

**DOI:** 10.1242/dev.157115

**Published:** 2018-02-01

**Authors:** Jessica R. Von Stetina, Laura E. Frawley, Yingdee Unhavaithaya, Terry L. Orr-Weaver

**Affiliations:** 1 Whitehead Institute, Cambridge, MA 02142, USA; 2 Department of Biology, Massachusetts Institute of Technology, Cambridge, MA 02142, USA

**Keywords:** *Drosophila*, Endocycle, Endomitosis, Subperineurial glia, Salivary gland, Polyploid

## Abstract

Regulation of cell size is crucial in development. In plants and animals two cell cycle variants are employed to generate large cells by increased ploidy: the endocycle and endomitosis. The rationale behind the choice of which of these cycles is implemented is unknown. We show that in the *Drosophila* nervous system the subperineurial glia (SPG) are unique in using both the endocycle and endomitosis to grow. In the brain, the majority of SPG initially endocycle, then switch to endomitosis during larval development. The Notch signaling pathway and the String Cdc25 phosphatase are crucial for the endocycle versus endomitosis choice, providing the means experimentally to change cells from one to the other. This revealed fundamental insights into the control of cell size and the properties of endomitotic cells. Endomitotic cells attain a higher ploidy and larger size than endocycling cells, and endomitotic SPG are necessary for the blood-brain barrier. Decreased Notch signaling promotes endomitosis even in the ventral nerve cord SPG that normally are mononucleate, but not in the endocycling salivary gland cells, revealing tissue-specific cell cycle responses.

## Abstract (precis)

**Highlighted Article:** In *Drosophila* brain lobes, Notch and the mitosis-activating phosphatase String regulate the switch of subperineurial glia from endocycle to endomitosis during larval development, with endomitotic cells attaining increased ploidy and size.

## INTRODUCTION

Regulation of cell size and thus tissue and organ size is crucial for proper body formation and function in development. In many developmental contexts the production of very large cells is achieved through polyploidy, an increase in DNA content (Orr-Weaver, 2015). Polyploidy permits cells to attain sizes much larger than can result from growth alone. In plants and animals, polyploidy generally results from one of two cell cycle variant classes: the endocycle or endomitosis (Frawley and Orr-Weaver, 2015). In the endocycle, DNA replication alternates with a gap phase in the absence of mitosis, yielding cells with a single polyploid nucleus. Initially most commonly referred to as endoreduplication, the term endocycle has been used widely in the field for the past few decades to emphasize the cyclic nature of DNA replication and the crucial role of conserved cell cycle regulators (Fox and Duronio, 2013; Edgar et al., 2014). Endomitosis classically was defined as mitosis occurring within an intact nuclear envelope. This occurs in a restricted number of cell types, typically found in insects (Nagl, 1978). We and others have extended this term to include all cell cycle variants in which some aspects of mitosis such as anaphase A or even nuclear division occur in the absence of cytokinesis (Fox and Duronio, 2013; Frawley and Orr-Weaver, 2015). Thus, whereas the endocycle produces mononucleate polyploid cells, endomitosis can produce mononucleate or multinucleate polyploid cells depending on the specific form of endomitosis. Although increased ploidy leads to increased cell size, it is unclear what distinctions or advantages single versus multiple nuclei could impart.

In mammals both the endocycle and endomitosis are used; for example, the placental trophoblast giant cells (TGCs) endocycle, whereas blood megakaryocytes (MgKs) endomitose. In TGCs, the increase in cell size driven by polyploidy is hypothesized to facilitate their function of providing a barrier between the maternal and embryonic tissues (Rossant and Cross, 2001; Cross, 2005; Watson and Cross, 2005). Polyploidy leads to an increase in MgK cytoplasmic volume that permits adequate platelet production (Ravid et al., 2002). In *Drosophila*, many differentiated cell types become polyploid via the endocycle. Moreover, replicated sister chromatids are held in register to produce polytene chromosomes with stereotypic banding patterns. Increases in ploidy facilitate robust gene expression, as in the *Drosophila* germline nurse cells that synthesize and deposit maternal stores into the developing oocyte (Spradling, 1993). Regulation of cell size by ploidy also dictates the size of anatomical structures produced by polyploid cells such as the bristles on the adult body (Salle et al., 2012).

Recently, our understanding of this repertoire was expanded by our identification of a role for polyploidy in the *Drosophila* nervous system. The subperineurial glia (SPG) cells in the *Drosophila* larval brain, a subset of surface glia, do not increase in number during development, but rather increase their size by polyploidization (Unhavaithaya and Orr-Weaver, 2012). The SPG are present throughout the nervous system: in the brain lobes, the ventral nerve cord (VNC) and the peripheral nerves (Limmer et al., 2014). SPG function both as the blood-brain barrier (BBB) and as a niche and energy metabolism center to control reactivation and division of the underlying neuroblasts (Bainton et al., 2005; Schwabe et al., 2005; Spéder and Brand, 2014; Bailey et al., 2015; Volkenhoff et al., 2015). Increased SPG cell size due to changes in ploidy is necessary to coordinate growth with increasing underlying neuronal mass in order to maintain the integrity of the BBB without disruption of the SPG envelope by cell division and cytokinesis (Unhavaithaya and Orr-Weaver, 2012). Interestingly, either decreases or increases in SPG ploidy lead to defects in the BBB (Li et al., 2017).

All of the previously characterized *Drosophila* tissues employ the endocycle to increase their ploidy and are mononucleate, with the exception of the binucleate cells of the male accessory gland (Edgar and Orr-Weaver, 2001; Taniguchi et al., 2012). The SPG are unique because in the brain two types of SPG cells are observed: mononucleate and multinucleate (Unhavaithaya and Orr-Weaver, 2012). Functional roles for these two SPG types are unknown, as is the cell cycle mechanism, developmental timing and regulation of their formation. The SPG provide the opportunity to investigate whether a specific cell type can undergo both the endocycle and endomitosis, to monitor the impact of these two variant cell cycles on increased cell size through cell ploidy, and to explore how signaling pathways affect the choice between the two.

## RESULTS

### Developmental cell cycle control in the SPG

The presence of both mononucleate and multinucleate cells in the SPG of the *Drosophila* third instar larval brain led us to hypothesize that two types of variant cell cycles lead to increases in SPG ploidy (Unhavaithaya and Orr-Weaver, 2012). Mononucleate SPG could result from an endocycle with solely gap and S phases, whereas multinucleate SPG could be the consequence of a form of endomitosis in which nuclear division occurs in the absence of cytokinesis. This is in contrast to the mononucleate SPG in the VNC and peripheral nervous system (PNS). Here, we tested the hypothesis that the SPG in the brain lobe undergo two types of variant cell cycles.

**Fig. 1.**
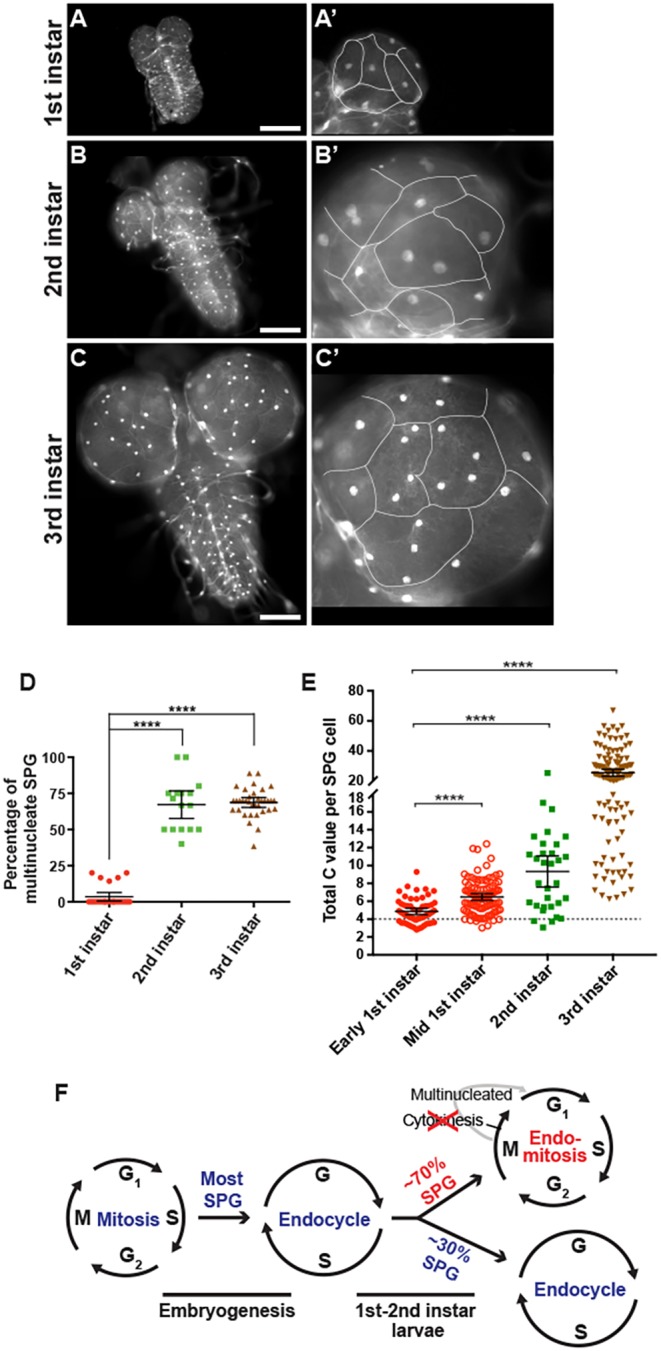
**Appearance of multinucleate SPG occurs between the first and second instar larval stages.** In this and all subsequent larval brain micrographs, the SPG nuclei are labeled with UAS-GFP^nls^ driven by *moody-GAL4* and shown in white or green. See Table S1 for complete genotypes for all figures. (A) Whole brain from first instar larva, with brain lobes predominantly containing mononucleate SPG. (B) Whole brain from second instar larva in which the majority of SPG are multinucleate. (C) Whole brain from wandering third instar larva. Both mononucleate and multinucleate SPG can be seen in the brain lobes. (A′-C′) Enlargements of the right brain lobe from A-C, respectively, with SPG outlines marked here (and in subsequent figures) by NRXIV-GFP highlighted in white. Scale bars: 100 μm in A-C. (D) Scatter plot showing the percentage of multinucleate SPG from *GAL4* driver-alone brains. First instar, *n*=130 SPG, 25 brains, two biological replicates; second instar, *n*=75 SPG, 16 brains, one biological replicate; third instar, *n*=375 SPG, 29 brains, two biological replicates. Of the 67% multinucleate SPG in second instar larvae, 86% were binucleate and 14% were tetranucleate. Kruskal–Wallis with Dunn's multiple comparisons test, ****P<0.0001. (E) Scatter plot showing DNA ploidy C values per SPG cell during larval development. Early first instars, 24-28 h AED, *n*=60 SPG, 19 brains; mid-first instars, 36-40 h AED, *n*=102 SPG, 19 brains; second instars, 60-64 h AED, *n*=32 SPG, 17 brains; third instars, wandering larvae, *n*=128 SPG, 58 brains. Data from first and second instars, one biological replicate; third instars, seven biological replicates. Mann–Whitney two-tailed test, ****P<0.0001. The *y*-axis contains a break and two different scales to better show the lower ploidy C values in early versus mid-first instars. Scatter plots show mean±95% confidence interval (c.i.). (F) Model for developmental cell cycle control in the SPG. Nearly all SPG transition from mitosis to the endocycle during embryogenesis. By the second instar larval stage, ∼30% of SPG remain in the endocycle, whereas ∼70% switch to endomitosis. The endocycle leads to polyploid cells with a single nucleus, whereas endomitosis leads to multinucleate cells.

We first investigated when these two types of SPG cells appear in development. It was previously shown that SPG cell number does not increase during the three larval instar phases but that SPG ploidy increases (Unhavaithaya and Orr-Weaver, 2012), but now we examined the temporal transition and ploidy of the mononucleate versus multinucleate cells. We dissected brains from first and second instar larvae in which SPG nuclei were labeled by UAS-GFP^nls^ driven by *moody*-*GAL4*. Mononucleate versus multinucleate SPG were distinguished by labeling the cell boundaries with Neurexin IV (NRXIV)-GFP, a component of septate junctions (Banerjee et al., 2010). In contrast to third instar larvae, in which ∼70% of brain SPG are multinucleate, nearly all of the first instar larvae were mononucleate. We identified only 4% in first instar brains that had multiple nuclei, and these had only two nuclei (Fig. 1A-D). The number of multinucleate SPG increased to 68% in second instar, with nearly all having two nuclei and a few having four nuclei. By third instar 18% had two, 35% had four, and 14% had eight nuclei (see Fig. 3D). We examined the position of multinucleate versus mononucleate SPG in the brain lobes by scoring their presence in the half of each lobe of the third instar brain adjacent to the VNC versus distal. We observed that 97% of brain lobes had mononucleate SPG located adjacent to the VNC, whereas only 3% of the lobes had mononucleate SPG located distally (Fig. S1A,B); 97% of scored mononucleate SPG cells themselves were found to be proximal to the VNC (Fig. S1B).

We next analyzed whether the mononucleate SPG present in first instar larvae were endocycling, as it was possible they were arrested in the mitotic cell cycle or in a G0 state. We measured the ploidy of the nuclei at two points in the first instar stage, and in the second and third instar stages. In early first instar larvae the SPG range between 2.8C and 9.3C, with a mean of 4.9C (Fig. 1E). The cells with a ploidy higher than 4C are most likely endocycling. This is supported by the finding that the ploidy of the cells increases to a range of 3C to 12.4C with a mean of 6.5C by mid-first instar, as well as the fact that we observed EdU incorporation in ∼12% of these SPG (Fig. 1E, Fig. S2A-A″). Finally, we tested whether the cells were in a mitotic state by staining with antibodies to the mitotic cell cycle markers Cyclin B and phospho-histone H3. The level of Cyclin B staining was uniformly low across the SPG cell layer in the first instar brain lobe (Fig. S3). Furthermore, none of the SPG examined showed detectable phospho-H3 staining (Fig. S2B-B″). We conclude from the increased ploidy, detectable DNA replication, and absence of mitotic markers that at least 93% of the SPG in the larval brain are in the endocycle by the mid-first instar stage. These cells most likely continue endocycling during larval development, as the percentage of mononucleate SPG stays constant after the second instar yet the ploidy of these cells increases from first to third instar larval stage (Fig. 1D,E, Fig. 6A).

Several lines of evidence indicate that the multinucleate cells appearing between the first and second instar result from endomitosis. First, the number of SPG cells does not change during development, and thus cell fusion does not occur (Unhavaithaya and Orr-Weaver, 2012). Second, the ploidy and number of nuclei in the multinucleate SPG increase during development (Fig. 1E, Fig. 3D). Third, we previously observed that SPG with multiple nuclei label with anti-phospho-H3 during larval stages (Unhavaithaya and Orr-Weaver, 2012). Fourth, and most importantly, the mitosis-activating Cdc25 phosphatase String (Stg) is required for multinucleate SPG.

**Fig. 2.**
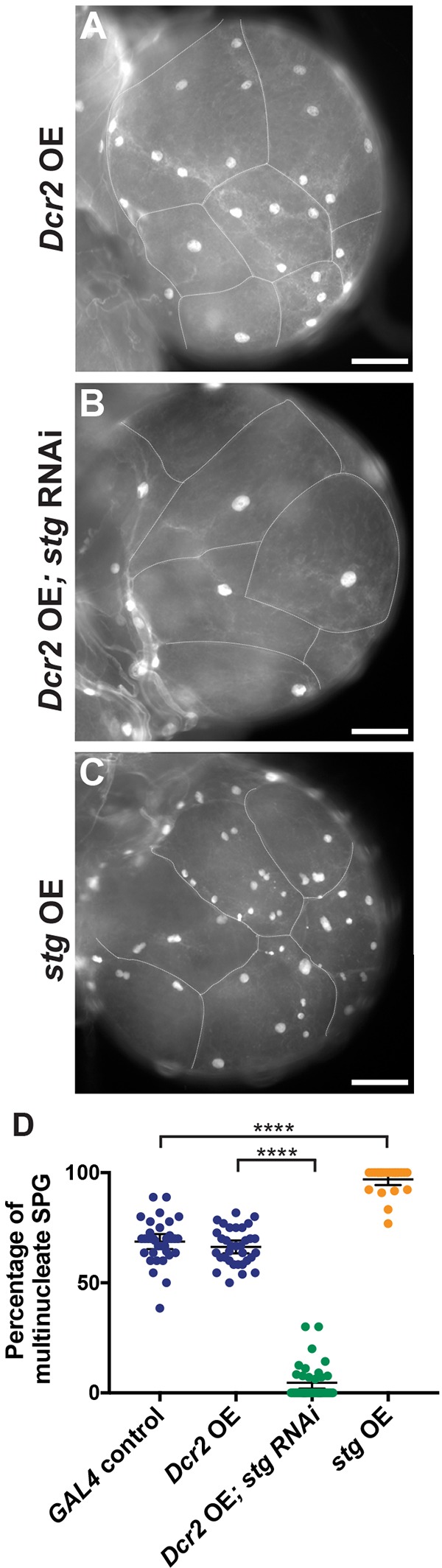
**Levels of the cell cycle phosphatase Stg affect endomitosis in SPG.** (A) Control brain lobe showing both endocycling (mononucleate) and endomitotic (multinucleate) SPG. (B) *Dcr2* overexpression (OE); *stg* RNAi brain lobe with only mononucleate SPG. (C) *stg* OE brain lobe with mostly endomitotic SPG. All brain lobes are from wandering third instar larvae. Scale bars: 100 µm. (D) The percentage of multinucleate SPG. *GAL4* control, *n*=375 SPG, 29 brains, two biological replicates; *Dcr2* OE, *n*=389 SPG, 19 brains, one biological replicate; *Dcr2* OE; *stg* RNAi, *n*=421 SPG, 19 brains, two biological replicates; *stg* OE, *n*=286 SPG, 13 brains, one biological replicate. Mann–Whitney two-tailed test, ****P<0.0001. Mean±95% c.i. Data for *GAL4* control are from Fig. 1D (third instar).

Stg is a crucial activator of mitosis in *Drosophila*, and we find that its levels control the number of SPG with multiple nuclei. It has been shown previously that mitotic Cyclin/CDK kinase is off and dispensable for the endocycle (Edgar et al., 2014). The Cdc25 phosphatase Stg is essential for active Cyclin B/CDK1 and mitosis in *Drosophila* but is repressed in the endocycle (Edgar and O'Farrell, 1990; Deng et al., 2001). Therefore, we evaluated the role of Stg and presumably active mitotic Cyclin/CDK in the appearance of multinucleate SPG by reducing its levels by RNAi specifically in the SPG with the *moody-GAL4* driver (Fig. S3J). Reduction of *stg* function dramatically inhibited the appearance of multinucleate SPG (Fig. 2A,B), causing 95% of third instar brain SPG to be mononucleate (Fig. 2D, Fig. S1). By contrast, overexpressing Stg specifically in the SPG increased the percentage of multinucleate SPG to 97% in the third instar brain (Fig. 2C,D, Fig. S1C-E, Fig. S3I,J), although it did not increase the percentage of multinucleate SPG in the first instar brain (5%, compared with 4% for the control). We also observed a significant increase in the percentage of mononucleate SPG in third instar brains [40% (*n*=499 SPG, 23 brains) compared with 31% (*n*=373 SPG, 20 brains) in the control, *P*=0.02] when *stg* RNAi was induced with the *Gliotactin-GAL4* driver (Schulte et al., 2003). Overexpression of *stg* with *Gliotactin-GAL4* also significantly increased the percentage of multinucleate SPG in third instar brains [99% (*n*=532 SPG, 23 brains) compared with 69% (*n*=373 SPG, 20 brains) in the control, *P*<0.0001]. The effect of changes in Stg expression on the percentage of mononucleate versus multinucleate SPG is consistent with our observation that Cyclin B protein is not detectable in first instar SPG, but the protein is present in second instar SPG in the brain lobes (Fig. S3). Taken together, these results indicate that mitosis and nuclear division occur in the SPG in the absence of cell division, a form of endomitosis that initiates between the first and second instar stages in 70% of the SPG (Fig. 1F). This proportion remains constant through the third instar, although the SPG ploidy continues to increase (Fig. 1D,E, Fig. 6A).

Collectively, the results on the mononucleate and multinucleate SPG are most simply explained by the SPG cells in the brain lobes becoming polyploid by two different cell cycle variants: the endocycle and endomitosis. Direct imaging of mitotic divisions in the brain SPG is not technically possible because it would not only require *ex vivo* culturing of the first instar larval brain for at least 24 h and up to 3 days but also producing hormonal shifts to mimic the larval molts; such culturing conditions have not been developed. Moreover, there are only ∼10 multinucleate SPG cells per brain lobe, and each of these undergoes only one nuclear division at some point within a 24 h window.

Because at least 93% of brain SPG are endocycling in mid-first instar brain lobes and 70% initiate endomitosis between first and second instar stages, we conclude that SPG cells can undergo two cell cycle transitions: from mitotic divisions to the endocycle late in embryogenesis or early first instar, and then from the endocycle to endomitosis at the end of first instar. Although we failed to detect phospho-H3 in mononucleate SPG we cannot exclude the possibility that these cells have some mitotic characteristics at some point during larval development, but the level of mitotic activity is insufficient for nuclear divisions.

### Notch signaling inhibits endomitosis

Given the dynamic cell cycle transitions occurring in the brain SPG, we investigated potential developmental regulators. Notch (N) signaling has been demonstrated to link cell cycle alteration to developmental events. It has been shown to trigger endocycle onset in *Drosophila* adult follicle and midgut cells (Deng et al., 2001; Lopez-Schier and St Johnston, 2001; Micchelli and Perrimon, 2006; Ohlstein and Spradling, 2006), but, in contrast, is necessary for the mitotic divisions that follow the two endocycles in the rectal papillae (Schoenfelder et al., 2014).

**Fig. 3.**
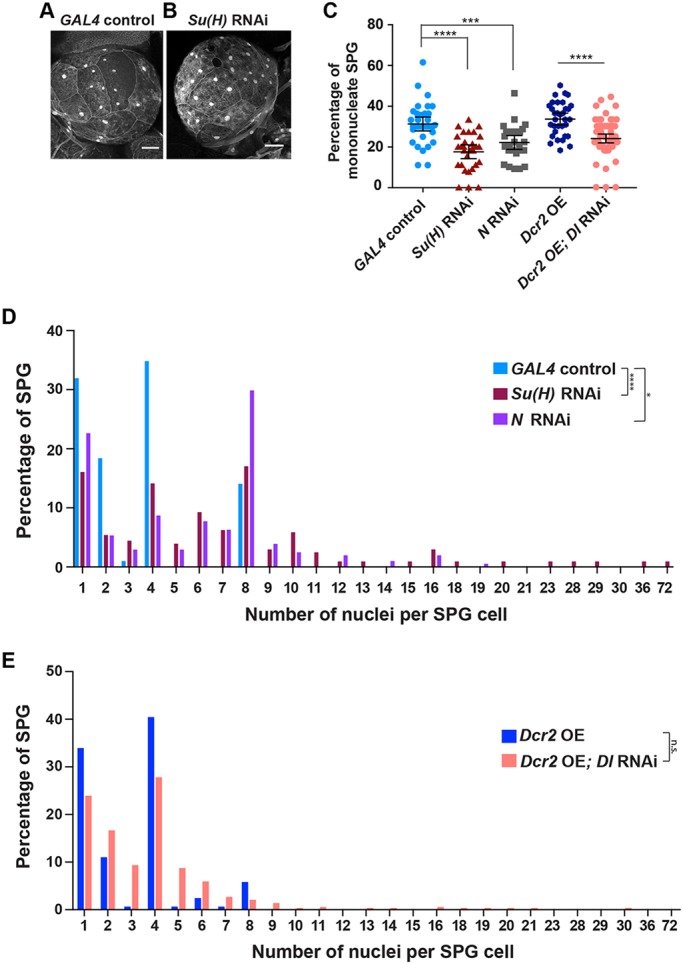
**Inhibition of Notch signaling leads to a shift from the endocycle to endomitosis and increased mitoses in endomitotic SPG.** (A) *GAL4* control brain lobe. (B) *Su(H)* RNAi brain lobe. Scale bars: 50 µm. (C) The percentage of mononucleate SPG. *Dcr2* OE is the control for *Dcr2* OE; *Dl* RNAi. *GAL4* control, *n*=375 SPG, 29 brains; *Su(H)* RNAi, *n*=312 SPG, 22 brains; *N* RNAi, *n*=313 SPG, 23 brains; *Dcr2* OE, *n*=389 SPG, 19 brains; *Dcr2* OE; *Dl* RNAi, *n*=670 SPG, 47 brains. *Dcr2* OE data, one biological replicate; all other data, two biological replicates. Kruskal–Wallis with Dunn's multiple comparisons test, ***P=0.0007, ****P<0.0001. Mean±95% c.i. The *Dcr2* OE data are the same as in Fig. 2D and the *GAL4* control data are the same as in Fig. 1D (third instar) and Fig. 2D. (D,E) The number of nuclei per SPG cell, displayed as a percentage of total SPG cells. (D) *GAL4* control, *n*=207 SPG, 38 brains; *Su(H)* RNAi, *n*=206 SPG, 25 brains; *N* RNAi, *n*=208 SPG, 17 brains, three biological replicates. Mann–Whitney two-tailed test, *P=0.037, ****P=0.0003. (E) *Dcr2* OE, *n*=320 SPG, 19 brains; *Dcr2* OE; *Dl* RNAi, *n*=466 SPG, 27 brains, one biological replicate. Mann–Whitney two-tailed test; not significant (n.s.), *P*=0.14.

We reduced the levels of Su(H), the transcription factor that is the major N signaling downstream effector (Bray, 2006), by RNAi in SPG cells, using the same experimental strategy described above for *stg*. We also reduced the N receptor by RNAi in the SPG under *moody-GAL4* control. We observed differences in the ratio of mononucleate to multinucleate SPG in *Su(H)* RNAi third instar brains when compared with control brains (Fig. 3A,B). Quantification revealed a significantly decreased number of mononucleate cells relative to multinucleate cells in *Su(H)* RNAi and *N* RNAi (Fig. 3C). A mean of 31% of control SPG were mononucleate, but only 18% of *Su(H)* RNAi SPG and 22% of *N* RNAi cells were mononucleate. Driving *Su(H)* RNAi with *Gliotactin-GAL4* also significantly reduced the percentage of mononucleate SPG in third instar brains [24% (*n*=413 SPG, 24 brains) compared with 31% (*n*=373 SPG, 20 brains) in the control, *P*=0.0004]. Therefore, similar to its role in blocking cell proliferation and promoting the endocycle in the adult follicle cells and midgut, our results indicate that N signaling acts to maintain the endocycle and inhibit endomitosis in the SPG. *Su(H)* RNAi did not significantly change the level of *stg* mRNA in the SPG (Fig. S3J), so the effect of N signaling on endomitosis in the SPG might not be via inhibition of *stg*.

The finding that N activity retains cells in the endocycle raised the question of the source of the ligand. To test whether signaling between SPG, such as lateral inhibition, might affect the endocycle versus endomitosis decision, we reduced Delta (Dl) levels in the SPG with RNAi driven by *moody-GAL4*. This also resulted in a significantly lower percentage of SPG undergoing endocycles (Fig. 3C). Thus, lateral inhibition between SPG in the brain lobe mediated by Dl-N interactions may retain some SPG in the endocycle. However, *moody-GAL4*-driven *Dl* RNAi occurred in all SPG, including those in the VNC that are solely mononucleate. Therefore, given that the endocycling brain SPG lie in the half of the brain lobe closest to the VNC, it is possible that Dl on the SPG in the VNC that are in direct contact with the brain lobe SPG might cause them to remain in the endocycle.

### Ablation of the Notch signaling pathway perturbs mitotic divisions during endomitosis

The SPG provided the opportunity to define the characteristics of endomitosis. Normally, an integral number of mitotic divisions occur, such that nearly all the cells contain two, four or eight nuclei (Fig. 3D). In addition to increasing the percentage of SPG cells undergoing endomitosis, the number of nuclear divisions was increased when Su(H), N, and Dl were perturbed. Up to 72 nuclei were observed in third instar brain SPG from *Su(H)* RNAi animals (Fig. 3D). RNAi against *N* also increased nuclear divisions, as shown by the percentage of SPG with eight nuclei being more than doubled compared with the driver-alone control (Fig. 3D). Moreover, cells with odd numbers of nuclei were present with all three methods of reducing N signaling (Fig. 3D,E). To date, no roles for Su(H) in cell cycle regulation independent of N have been described. Thus, the greater effect of *Su(H)* RNAi on nuclear number over *N* RNAi could be due to differential knockdown levels or to differences in the threshold levels required for activation of the signaling pathway between Su(H) and N.

The increased nuclear number observed when N signaling was reduced was associated with a significant decrease in the ploidy of individual nuclei (Fig. S4A,B). Notably, nuclei with less than a 2C genomic content were present when N signaling was perturbed (Fig. S4B). Whereas only 1.4% of nuclei quantified in control endomitotic SPG cells had a C value of less than or equal to a diploid 2C, this percentage increased to 18% in *Su(H)* RNAi endomitotic SPG (Fig. S4B). The nuclei with less than a diploid DNA content could result from errors in mitotic chromosome segregation, as we observe anaphase bridges, or perhaps from mitosis occurring in the absence of completed DNA replication. Problems separating polytene chromosomes in mitosis also could account for cells with less than 2C content.

### SPG in the VNC, but not salivary gland cells, are capable of endomitosis

*Drosophila* polyploid rectal papillar cells are able to return to mitotic divisions after endocycling (Fox et al., 2010), the only tissue to date observed to switch out of the endocycle during *Drosophila* development. Given the striking promotion of endomitosis by the reduction of Su(H) function in polyploid SPG cells, we investigated whether other larval endocycling tissues retain the ability to enter mitosis.

**Fig. 4.**
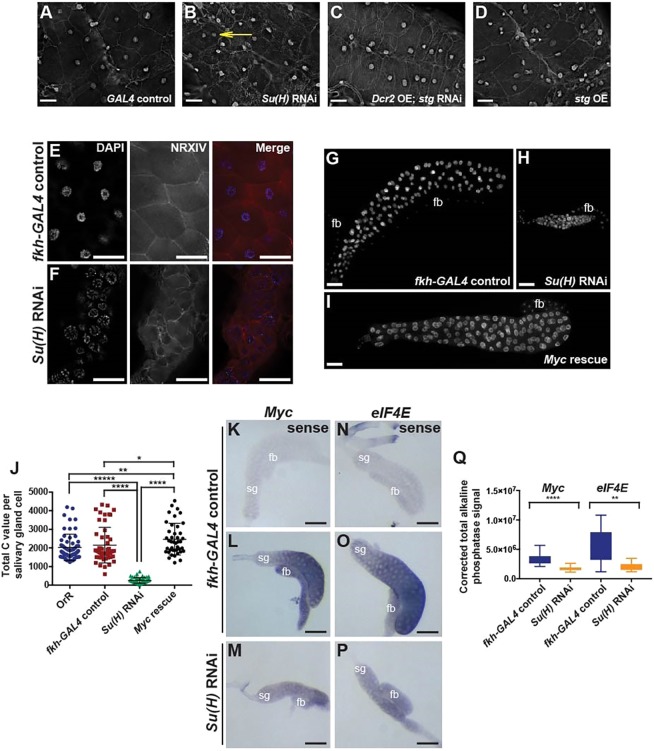
**Endocycling** **SPG of the VNC are** **susceptible** **to enter endomitosis, but salivary gland endocycling cells are not.** (A-D) VNCs from *GAL4* control (A), *Su(H)* RNAi (B), *Dcr2* OE; *stg* RNAi (C) and *stg* OE (D) wandering third instar larvae. Yellow arrow (B) points to a binucleate SPG. See also Table 1. (E,F) High-magnification images of salivary glands from *fkh-GAL4*-alone control (E) and *Su(H)* RNAi (F) stained with DAPI or NRXIV antibody, and merge. NRXIV staining shows the cell boundaries, revealing all the salivary gland cells in *Su(H)* RNAi larvae to be mononucleate. (G-I) DAPI-stained salivary glands from wandering third instar larvae. Control *fkh-GAL4* alone (G), *Su(H)* RNAi (H) or *Su(H)* RNAi; *UAS-Myc* rescued (I) salivary glands. fb, fat body. (J) DNA ploidy values from wandering third instar salivary gland nuclei. Control OrR, *n*=58 nuclei, 9 salivary glands; *fkh-GAL4*-alone control, *n*=53 nuclei, 10 salivary glands; *Su(H)* RNAi, *n*=50 nuclei, 8 salivary glands; *Myc* rescue, *n*=41 nuclei, 8 salivary glands, one biological replicate. Mann–Whitney two-tailed test with Bonferroni adjustment, *P=0.033, **P=0.0053, ****P=8.2×10^−26^, *****P=6.9×10^−31^. Mean±95% c.i. (K-P) Alkaline phosphatase *in situ* hybridization revealed a reduction in transcript levels of *Myc* (L) and *eIF4E* (O) in *Su(H)* RNAi salivary glands (M,P). Probes homologous to the sense strands were used as a control (K,N). sg, salivary gland; fb, fat body. (Q) Corrected total alkaline phosphatase signals (arbitrary units) for *Myc* (*fkh-GAL4*-alone control, *n*=19 salivary glands; *Su(H)* RNAi, *n*=25 salivary glands) and *eIF4E* (*fkh-GAL4*-alone control, *n*=11 salivary glands; *Su(H)* RNAi, *n*=11 salivary glands) transcripts from single scanned images for each genotype, two biological replicates. Mann–Whitney two-tailed test, **P=0.0024, ****P<0.0001. Scale bars: 25 µm in A-D; 50 µm in E,F; 100 µm in G-I,K-P.

The SPG in the VNC endocycle: they increase ploidy throughout larval development, incorporate EdU, and yet remain mononucleate and lack phospho-H3 staining (Unhavaithaya and Orr-Weaver, 2012). We investigated whether perturbing N signaling or overexpressing the Stg Cdc25 phosphatase could cause SPG in the VNC to undergo endomitosis, scoring the number of larvae with at least one multinucleate cell in the VNC (Fig. 4A-D, Table 1). RNAi against *Su(H)* or *N* led to SPG in the VNC with multiple nuclei, as did overexpression of the *stg* gene. These multinucleate cells were readily detectable by third instar, but also had begun to appear by the first instar for *N* RNAi and *stg* overexpression (Table 1). Multinucleate cells in the first instar VNC, however, were rare, with animals having only one or two such cells. Thus, we scored by animal rather than SPG cell number, and we consider that the differences in percentage of multinucleation after *N* knockdown between first and third instar are unlikely to be biologically significant. We conclude that although SPG in the VNC normally do not undergo endomitosis they retain the capability of doing so if mitotic Cyclin/CDK is activated.

**Table 1.**
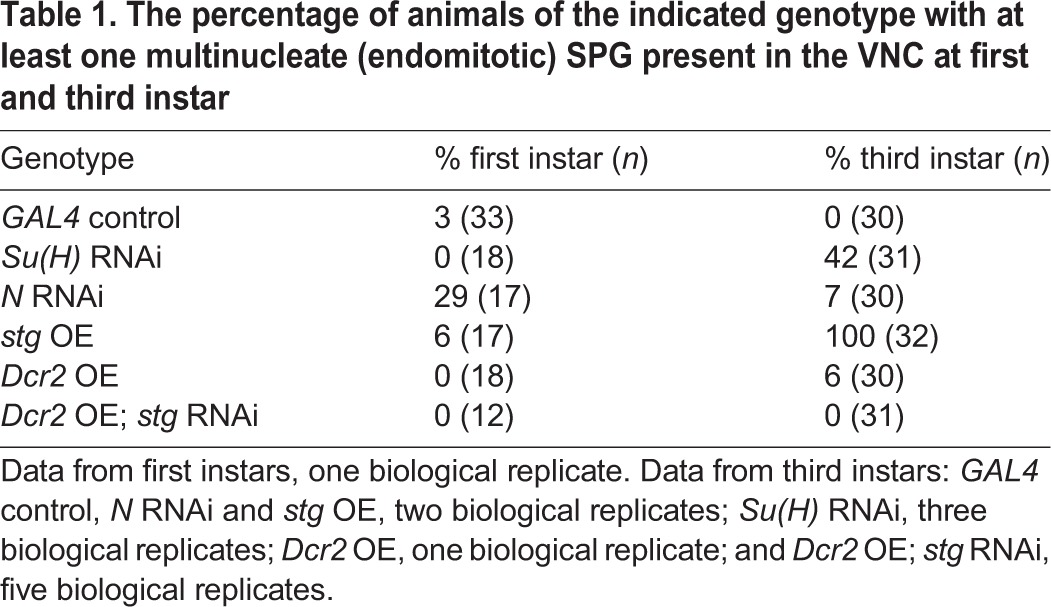
**The percentage of animals of the indicated genotype with at least one multinucleate (endomitotic) SPG present in the VNC at first and third instar**

By contrast, the endocycling cells of the salivary gland did not undergo endomitosis when N signaling was reduced, and Su(H) appears to play a distinct role in this tissue. We used the *forkhead* (*fkh*)*-GAL4* driver (Henderson and Andrew, 2000) to express *Su(H)* RNAi specifically in salivary glands beginning from when they first enter the endocycle during embryogenesis. Salivary glands were dissected from wandering third instar larvae, fixed, and stained with DAPI to visualize DNA. In contrast to SPG, we did not observe any salivary gland cells with more than one nucleus (Fig. 4E,F) and also did not detect staining for the mitotic marker phospho-H3 (data not shown). The failure of *Su(H)* RNAi to drive endocycling cells in the salivary gland into endomitosis is correlated with low levels of Cyclin B protein, although the SPG in the VNC also have low levels (Fig. S3C,F,H).

Reduction of N signaling did not result in endomitosis in the salivary gland, in contrast to the SPG, yet *Su(H)* RNAi expression in this tissue did affect ploidy and growth (Fig. 4G-J). Whereas the mean ploidies for wild-type OrR and *fkh-GAL4* control salivary glands were 2031C and 2148C, respectively, *Su(H)* RNAi salivary glands had a mean ploidy of 256C (Fig. 4J). EdU incorporation measurements showed that this correlated with a reduction of the number of cells in S phase (Fig. S5A-F,J). Analysis of polytene chromosome spreads from salivary glands also revealed strong defects in chromosome structure accompanying *Su(H)* RNAi (Fig. S5K-Q).

A connection between N signaling through Su(H) and Myc-mediated cell growth was found in *Drosophila* neural stem cells (NSCs) (Song and Lu, 2011). It was shown that Su(H) directly activates *Myc* transcription, which in turn increases mRNA expression of the translation initiation factor *eIF4E* to promote cell growth and subsequent maintenance of NSC fate. Given these findings, we asked whether a similar N-mediated regulation of zgrowth occurs in the salivary glands. We analyzed *Myc* and *eIF4E* mRNA levels in salivary glands from 96-120 h after egg deposition (AED) larvae by alkaline phosphatase *in situ* hybridization. We found that, relative to *fkh-GAL4* controls, *Su(H)* RNAi salivary glands had decreased levels of *Myc* and *eIF4E* mRNAs (Fig. 4K-Q).

These findings suggested that Myc is a key downstream effector of Su(H) signaling in regulating growth in salivary glands, prompting us to test whether its overexpression might be able to rescue the *Su(H)* RNAi phenotypes. Indeed, the cell size, ploidy, and EdU incorporation defects resulting from *Su(H)* RNAi were fully rescued by *Myc* overexpression (Fig. 4G-J, Fig. S5G-J). Our results argue that, in contrast to the SPG where Su(H) and N signaling regulate the choice between the endocycle and endomitosis, in the salivary gland Su(H) solely controls the endocycle, regulating growth and ploidy via Myc.

### The presence of both endocycling and endomitotic SPG is required for the BBB

The developmentally triggered onset of endomitosis in the SPG and failure of salivary gland cells to undergo endomitosis when Su(H) was reduced raised the issue of the function and requirement for endomitotic SPG. The key role of SPG is to form the BBB through septate junctions (Stork et al., 2008), and we have shown that ploidy is crucial to increase SPG cell size as the nervous system develops while preserving intact septate junctions (Unhavaithaya and Orr-Weaver, 2012). The effect of perturbation of N signaling or changes in the levels of Stg permitted us to evaluate the role of endomitosis in the BBB, as decreased N signaling increased the percentage of endomitotic SPG, reduction of Stg converted the brain to nearly entirely endocycling SPG, and overexpression of Stg caused nearly all SPG in the brain to endomitose.

**Fig. 5.**
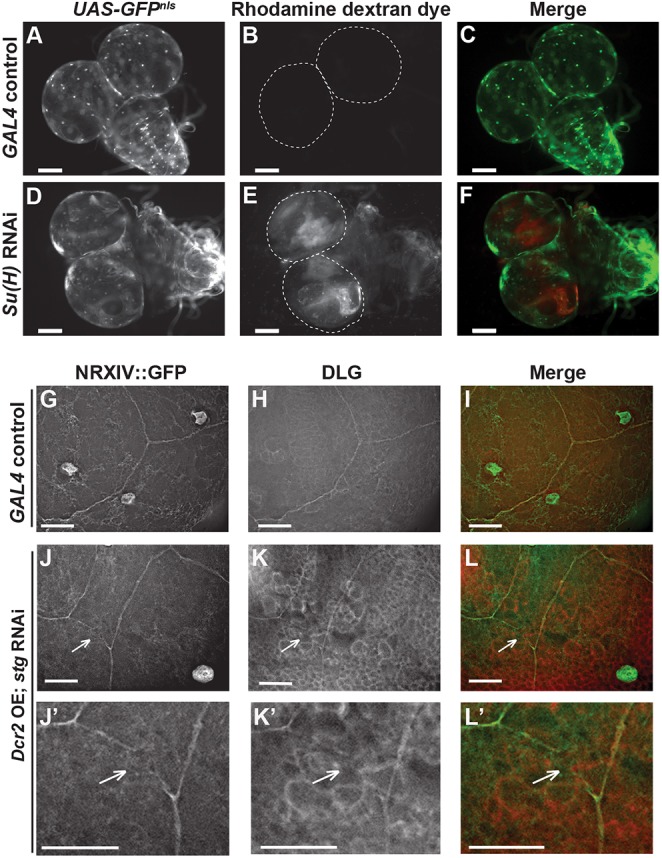
**The BBB** **becomes defective when the percentage of endomitotic SPG is altered.** Dye penetration assays were performed in brains of wandering third instar larvae. (A-F) No dye signal was detected inside the lobes of driver-alone control brains (A-C)*.* By contrast, the injected dye penetrated *Su(H)* RNAi brains (D-F)*.* The dashed lines in the Rhodamine-dextran panels mark the edges of the brain lobes. See also Table 2. (G-L) Analysis of the septate junction markers NRXIV and DLG in the brain lobes of wandering third instar larvae. (G-I) No visible septate junction breaks were detected in *GAL4* control brains. (J-L) Visible NRXIV and DLG breaks were detected in *Dcr2* OE; *stg* RNAi brains. (J′-L′) Enlargement of septate junction breaks in J-L. White arrows point to breaks. For *n* and *P* values see Fig. S6. Scale bars: 100 µm in A-F; 25 µm in G-L′.

We measured functionality of the BBB by injecting Rhodamine-conjugated dextran into the third instar larval body cavity of control, RNAi and overexpression animals and then scoring the percentage of brains into which the dye penetrated. Whereas dye penetrated the BBB in only 2% of *moody-GAL4*-injected larvae, dye penetration was detected in 69% of *Su(H)* RNAi, 29% of *N* RNAi, and 50% of *stg* overexpression brain lobes (Fig. 5, Table 2, Fig. S6). RNAi against *stg* also affected the BBB, as the dye penetrated 52% of brain lobes (Fig. 5, Table 2, Fig. S6). The RNAi depletion experiments for *stg* were performed in the presence of Dicer2 (Dcr2) to enhance knockdown. Induction of *Dcr2* alone affects the BBB, as 20% of brains in *UAS-Dcr2; moody-GAL4* showed dye penetration (Table 2). Nevertheless, depletion of *stg* showed a percentage of brains with dye penetration significantly higher than this control (Table 2).

**Table 2.**
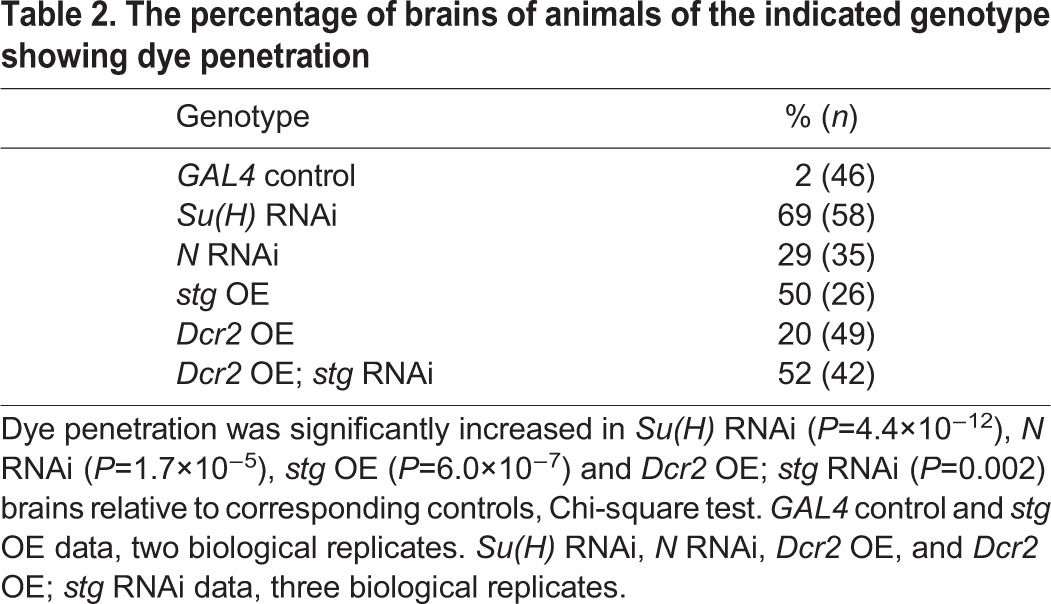
**The percentage of brains of animals of the indicated genotype showing dye penetration**

Blocking polyploidization of SPG by inhibiting DNA replication led to reduced cell size, BBB defects and ruptured intercellular septate junctions (Unhavaithaya and Orr-Weaver, 2012). The results above showed that either increasing or decreasing the proportion of endomitotic SPG caused BBB defects. To determine whether this could be due to alterations of the SPG cell envelope, we visualized septate junctions by staining with an antibody to the Discs large 1 (DLG) protein as well as with the NRXIV-GFP fusion (Fig. 5G-L). RNAi against *Su(H)* and *stg*, and overexpression of *stg*, all resulted in breaks in septate junction tracks that were visible with both protein markers, in contrast to driver-alone controls (Fig. 5G-L, Fig. S6M). Breaks in the septate junctions were significantly increased in *Su(H)* and *Dcr2* overexpression; *stg* RNAi brains. Although breaks were elevated following Stg overexpression, the significance was borderline. These results are consistent with the BBB defects resulting from breakage of the septate junctions.

### The relationship between ploidy, nuclear number and cell size in the endocycle versus endomitosis

Given that the presence of endomitotic SPG is crucial for the BBB, we compared how endomitosis and the endocycle affect cell size, total cell ploidy, and shape. The SPG tissue layer has to maintain a seal around the proliferating neuronal mass of the brain during larval development; consequently, SPG cell size is critical. One possibility is that endocycling versus endomitotic SPG have a different shape and that the right combination of these shapes enables them to cover and seal each brain lobe. We noted, however, that there is not a difference in cell shape arising from the two cell cycles (data not shown).

**Fig. 6.**
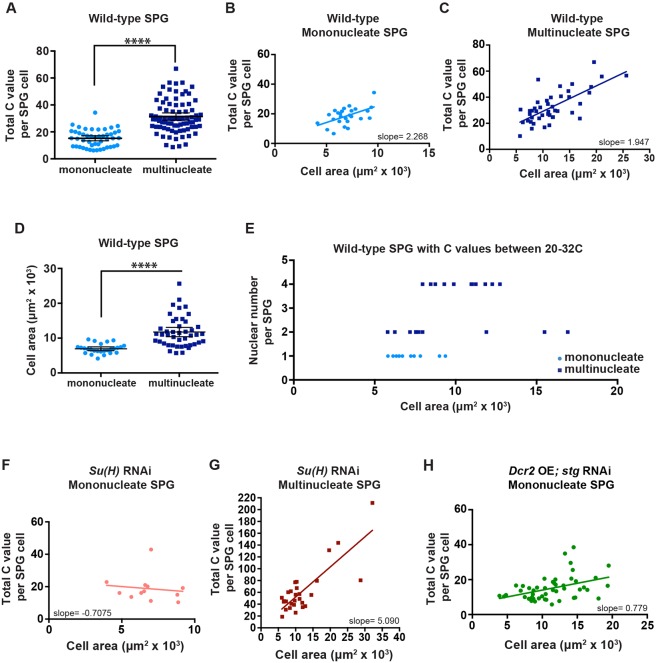
**Endomitotic cells are larger than endocycling cells.** (A) DNA ploidy C values of individual mononucleate and multinucleate SPG in the brain lobe. Mononucleate, *n*=47 SPG, 41 brains; multinucleate, *n*=81 SPG, 58 brains, seven biological replicates*.* Data are the same as in Fig. 1E (third instar). Mann–Whitney two-tailed test, ****P<0.0001. (B,C) DNA ploidy C values of mononucleate or multinucleate SPG as a function of cell area with linear regression. (B) Mononucleate, *n*=26 SPG, 24 brains, three biological replicates. Spearman correlation coefficient *r*=0.47, *P*=0.015; slope=2.268 C value/μm^2^×10^3^, *P*=0.0053. (C) Multinucleate, *n*=44 SPG, 33 brains, three biological replicates. Spearman correlation coefficient *r*=0.60, *P*<0.0001; slope=1.947 C value/μm^2^×10^3^, *P*<0.0001. (D) Cell area of individual mononucleate or multinucleate SPG in the brain lobe. Mononucleate, *n*=26 SPG, 24 brains; multinucleate, *n*=44 SPG, 33 brains; three biological replicates each. Mann–Whitney two-tailed test, ****P<0.0001. (E) Cell area in relation to SPG nuclear number. Only cells having a ploidy between 20C and 32C are plotted. *n*=31 SPG; 23 brains, three biological replicates. Spearman correlation coefficient *r=*0.60, *P*=0.0004. The ploidy values and size for each cell are given in Table S2. (F,G) Plots showing DNA ploidy C values of mononucleate or multinucleate SPG as a function of cell area with linear regression. (F) *Su(H)* RNAi mononucleate, *n*=12 SPG, 12 brains, three biological replicates. Spearman correlation coefficient *r*=−0.18, *P*=0.57; slope=−0.7075 C value/μm^2^×10^3^, *P*=0.68. (G) *Su(H)* RNAi multinucleate, *n*=28 SPG, 15 brains, three biological replicates. Spearman correlation coefficient *r*=0.54, *P*=0.003; slope=5.090 C value/μm^2^×10^3^, *P*<0.0001. (H) *Dcr2* OE; *stg* RNAi mononucleate, *n*=48 SPG, 15 brains, one biological replicate. Spearman correlation coefficient *r*=0.42, *P<*0.003; slope=0.779 C value/μm^2^×10^3^, *P*<0.003. Scatter plots (A,D) mean±95% c.i. All samples were collected from wandering third instar larvae.

We examined whether total cell ploidy differed between endocycling and endomitotic SPG. As noted above, endomitotic SPG have up to eight nuclei, with four being the most frequent. We quantified DAPI fluorescence intensity in all of the nuclei in each SPG cell labeled with GFP^nls^, marking the cell boundaries with NRXIV-GFP. There was a significant difference: endocycling cells had a mean ploidy of 15C, whereas endomitotic cells had a mean ploidy of 31C (Fig. 6A).

We next examined how SPG cell size correlated with ploidy in the two variant cell cycles. SPG are flat cells of depth less than 1 µm, and so their area reflects cell size (Stork et al., 2008; Limmer et al., 2014). As expected, cell size scaled with total cellular DNA content in both cell cycle types (Fig. 6B,C). The mean area of the population of endomitotic SPG was larger than that of the endocycling SPG (Fig. 6D), consistent with their increased ploidy levels.

In addition to increased ploidy causing increased cell size, we examined whether increased nuclear number might also contribute to the increased size of endomitotic SPG. We compared cell size with nuclear number for endocycling and endomitotic SPG with different ploidies (Fig. S7A) as well as within a ploidy range of 20-32C (Fig. 6E). Nuclear number indeed augments cell size, as cells with four nuclei were larger than cells of the same ploidy with one or two nuclei (Table S2). We conclude that both ploidy and nuclear number contribute to SPG cell size, and that endomitosis is likely to be a mechanism to increase cell size in brain lobes to maintain the BBB. This is consistent with the smaller SPG cell size in the VNC and absence of endomitosis in these cells.

We examined cell size in the *stg* and *Su(H)* RNAi lines to test whether the higher ploidy associated with endomitotic cells compared with endocycling cells persisted with these perturbations, and also to investigate whether cell sizes were altered. As expected, in *stg* RNAi; *Dcr2* overexpression SPG, which were nearly all endocycling, the ploidy was equivalent to wild-type endocycling SPG, but reduced compared with wild-type endomitotic SPG (Fig. S7B). The *Su(H)* RNAi SPG that were multinucleate and had higher nuclear numbers than wild-type multinucleate SPG correspondingly had higher ploidy levels (Fig. S7C). Unexpectedly, in both of these RNAi conditions the normal positive link between ploidy and cell size became uncoupled, at least by the third instar stage when cell size was measured (Fig. 6F-H, Fig. S7D,E). When either Su(H) or Stg was reduced, mononucleate cells of the same ploidy exhibited a greater range of sizes (Fig. 6F,H), and cells of the same size exhibited a greater range of ploidy (especially the multinucleate *Su(H)* RNAi cells, Fig. 6G). Thus, the defects in the BBB might not be due solely to an alteration in SPG cell size.

## DISCUSSION

*Drosophila* SPG cells illustrate the developmental strategy of employing variant cell cycles for organogenesis. Here we find that SPG in the brain lobe can undergo two cell cycle changes during development, initiating the endocycle but reinstating nuclear division to increase nuclear number during endomitosis. The promotion of mitosis requires the Stg Cdc25 phosphatase and is limited by N signaling. Importantly, the presence of endomitotic SPG is essential for the BBB. These results support the conclusion that endomitosis and increased nuclear number are a mechanism for further increases in cell size beyond those attained by the endocycle.

### The role and regulation of endomitosis in the SPG

Despite the prevalence of polyploidy throughout the plant and animal kingdoms and the consistent correlation between ploidy and cell size, it remains a mystery why size scales with ploidy. We found that endomitotic cells are larger than endocycling cells, but this is due only in part to their higher ploidy. Interestingly, increased nuclear number also contributes to increased cell size. The SPG are flat cells, and the localization of the nuclei throughout the cell in endomitotic SPG could facilitate better distribution of gene products than in endocycling cells, in which large regions of the cytoplasm are distant from the nucleus. The properties of SPG in a brain tumor mutant are consistent with the use of endomitosis and increased nuclear number augmenting cell size. We previously found that in a tumor mutant with unregulated neuroblast cell divisions, the SPG respond by increasing their ploidy, thereby preserving the BBB (Unhavaithaya and Orr-Weaver, 2012). We note that this is associated with an increased nuclear number in the endomitotic SPG cells, likely permitting a further size increase in the SPG in response to the increased underlying neuronal mass.

Two unexpected findings that remain to be understood mechanistically are: (1) endomitotic cells of a given ploidy can exhibit a range of cell sizes; and (2) perturbation of N signaling or cell cycle regulation can uncouple the relationship between size and ploidy. It might be that gene expression is not uniform across the multiple nuclei of an endomitotic cell, resulting in variance of growth and cell size with respect to nuclear number. Signals from surrounding cell layers may differentially control the growth of wild-type endomitotic SPG and could promote the growth of *stg* RNAi endocycling SPG such that by the third instar stage they are able to attain the size normally associated with the endomitotic cells of increased ploidy.

The active developmental control of the onset of endomitosis in the SPG between first and second larval instar will make it important in the future to identify the developmental cues promoting the onset of endomitosis and distinguishing which SPG cells remain in the endocycle. Given the requirement for both N and Dl in the SPG for endocycling cells, it is possible that lateral inhibition causes some cells to endocycle and others to endomitose. This explanation, however, is not easily reconcilable with the spatial distribution of the two classes of SPG in the brain. Another possibility is that the Dl ligand is present in SPG in the VNC, causing them and the brain SPG closest to the VNC to remain in the endocycle.

In addition to defining developmental signals, questions remain about cell cycle control. The mechanism through which N signaling ensures both the regulation of nuclear division at a cellular level and the accuracy of mitosis remains to be defined. It is unclear whether N signaling can directly repress *stg* in the SPG. Reduction of Su(H) did not alter levels of *stg* mRNA, but Stg activity might be controlled post-translationally, because the Cdc25 phosphatase is known to be controlled by phosphorylation (Myer et al., 2005). Such regulation could explain why Cyclin B and Stg levels appear the same in endocycling and endomitotic SPG, despite the genetic experiments showing that perturbation of *stg* levels impacts the percentage of SPG in endomitosis.

### Ensuring a functional BBB

We found that the BBB is defective in the absence of endomitotic SPG following *stg* RNAi. A key question is whether the BBB defect is primarily a consequence of cell cycle changes. In *Drosophila*, the *stg* gene has not been observed to have functions or effects outside of cell cycle regulation, and thus the simplest explanation is that endomitosis itself is required for SPG function in the BBB. As discussed above, we hypothesize that endomitosis and the presence of multiple nuclei affect gene expression. The SPG subjected to *stg* RNAi have reduced ploidy compared with endomitotic SPG, and this also could impact gene expression. Because the *stg* RNAi cells can grow to nearly endomitotic size, the BBB defects might not be due primarily to cell size. The results here agree with previous work demonstrating that a functional BBB is dependent on proper SPG ploidy: either increases or decreases in ploidy lead to BBB defects (Unhavaithaya and Orr-Weaver, 2012; Li et al., 2017). In these latter studies ploidy was decreased by inhibiting DNA replication or increased in response to increased levels of Yorkie and Cyclin E and thus, as in the case of changing Stg levels, cell cycle changes led to BBB defects. It will be interesting to determine why SPG function is so sensitive to correct ploidy.

Given the many roles that N signaling can play in development, it may regulate SPG in multiple ways. Thus, when N signaling is reduced not only the ratios between the cell cycle variants but also differentiation of the SPG may be affected, leading to BBB defects. The defective BBB is unlikely to result from reduced expression of septate junction components, however, given that Su(H) has not been observed to bind to the regulatory regions of any of the genes that encode septate junction proteins (Contrino et al., 2012; Housden et al., 2013; Terriente-Felix et al., 2013; Slattery et al., 2014; Skalska et al., 2015).

### Developmental control of the cell cycle

We note several other cases in which cell cycle changes affecting cell differentiation and function have been investigated. A recent study in megakaryocytes shows that these cells remain functional to make platelets when switched from endomitosis to the endocycle (Trakala et al., 2015), in contrast to our results with *stg* RNAi demonstrating that endomitotic SPG are essential. In *C. elegans*, a group of intestinal cells undergoes one round of endomitosis under CDC-25 control to become binucleate at the end of the first larval stage. CDC-25 is then targeted for destruction, and the cells subsequently endoreduplicate (Lee et al., 2016). In mammalian hepatocytes the E2F7 and E2F8 transcription factors are required for polyploidization, but when they are knocked out hepatocyte differentiation and liver regeneration do not appear to be affected (Pandit et al., 2012). Thus, in this case, polyploidy might not play a crucial role.

The work presented here highlights distinct cell cycle capabilities in development and differentiation. In contrast to brain SPG, the SPG in the VNC do not normally undergo endomitosis yet are susceptible to change from the endocycle to endomitosis. Loss of Su(H) in the salivary gland, however, did not cause a return to mitosis. Myc is not the essential downstream target of Su(H) in the SPG, as overexpression of Myc does not rescue the endomitosis defects (data not shown). By contrast, *Su(H)* RNAi revealed a crucial role for Su(H) in promoting increased ploidy in the endocycle and thus growth in the salivary gland by regulation of Myc levels. These results illustrate the developmental plasticity of the cell cycle. Although the endocycle increases cell size, endomitosis results in larger, higher ploidy cells that are required in the brain lobe SPG. Thus, endomitosis appears to be a cell cycle variant that can be employed to meet extreme demands for cell growth and gene expression during development and organogenesis.

## MATERIALS AND METHODS

### Fly strains

*Drosophila* strains used were: Oregon R; *pUdsGFP-Su(H)* RNAi (gift from Anette Preiss, University of Hohenheim); *fkh-GAL4* (*III*) (provided by Deborah Andrew, Johns Hopkins University); *UAS-Myc* (*II*) (BDSC, #9674); *moody-GAL4* (*II*) (provided by Christian Klämbt, University of Münster); *NrxIV::GFP* (Banerjee et al., 2010); *UAS-N* RNAi (*II*) (VDRC, #1112); *UAS-CycB* RNAi (BDSC, #34544); *UAS-Dl* RNAi (MR182, II; gift from Matthew Rand, University of Rochester Medical Center); *UAS-Dcr2* (*X*) (VDRC, #60010); *UAS-stg* RNAi (*III*) (VDRC, #17760; gift from Don Fox, Duke University School of Medicine); and *UAS-stg* (BDSC, #4778).

### Antibodies

Primary antibodies were mouse anti-CycB [Developmental Studies Hybridoma Bank (DSHB), F2F4; 1:15] and anti-DLG (DSHB, 4F3; 1:15), rabbit anti-NRXIV (Manzoor Bhat, University of Texas Health Science Center; 1:1000), anti-phospho-histone H3 (Ser10; EMD Millipore, #06-570; 1:400), rat anti-ELAV (DSHB, 7E8A10; 1:15) and guinea-pig anti-GFP (gift from Mary-Lou Pardue, Massachusetts Institute of Technology; 1:400). The GFP-Booster nanobody (Chromotek, #gba488) was used at 1:400. Secondary antibodies were Alexa Fluor 568 goat anti-mouse or anti-rabbit (1:1000; Life Technologies), Alexa Fluor 647 goat anti-rabbit (1:1000; Life Technologies), and RRX- and Cy5-conjugated to donkey/goat anti-rabbit or anti-rat (1:250; Jackson ImmunoResearch).

### Dissection, fixation and antibody staining of larval tissues

Larval brains and salivary glands were dissected in either unsupplemented Grace's medium or PBS, fixed in 4% formaldehyde in PBS for 30 min, washed in PBST (0.3% Triton X-100) and blocked for 1 h at room temperature with blocking solution (PBST, 2.5% goat and 2.5% donkey serum). The methods for salivary gland polytene squashes, as well as for EdU and phospho-histone H3 labeling of salivary glands and larval brains are provided in the supplementary Materials and Methods.

### Mononucleate versus multinucleate SPG ratios

To determine SPG nuclear number we outlined the cell boundaries of wandering third instar larval brains using either NRXIV antibody or the NRXIV-GFP fusion protein. Mononucleate versus multinucleate SPG were scored after 3D reconstruction was performed using a Zeiss 710 confocal microscope with an LD LCI Plan-Apochromat 25×/0.8 Imm Korr DIC M27 objective and analyzed using ZEN software (Zeiss) for eight *GAL4* control, *Su(H)* RNAi and *N* RNAi brain lobes. For the remaining *GAL4* control, *Su(H)* RNAi and *N* RNAi brain lobes and all *Dcr2* overexpression; *Dl* RNAi brains, whole lobes were imaged using a Nikon Eclipse inverted microscope. For first and second instar larvae, images of half lobes from the Nikon Eclipse were used. To quantify the percentage of mononucleate versus multinucleate SPG, *z*-stacks and/or single frames of the dorsalmost and/or ventralmost surfaces of the brain were analyzed. To score all SPG, we demanded that the full outline of the SPG cell was visible in the NRXIV-GFP channel either in single scanned images or in multiple focal planes from *z*-stacks. Thus, any out of focus nuclei seen in the images are from the opposite side of the brain. Consequently, the data are likely to be an underestimate of cells with higher nuclear number.

To assess the multinucleate SPG present in the VNC, both the dorsal and ventral sides of the VNC were imaged on the Nikon Eclipse. Cells were scored as multinucleate only if the entire cell boundary was visible and consequently are likely to be an underestimate. An animal was scored as positive if any multinucleate cells were observed in the VNC.

To determine statistical significance, the Kruskal–Wallis test with Dunn's multiple comparisons or the Mann–Whitney two-tailed test was applied using GraphPad Prism. A Chi-square test was performed to determine statistical significance for differential localization of mononucleate versus multinucleate SPG proximal to the VNC.

### DAPI staining/ploidy C value calculations

Samples were stained with DAPI at 50 ng/ml, the optimal concentration for quantification of DNA ploidy (Hamada and Fujita, 1983), washed overnight, and imaged using the Nikon Eclipse inverted microscope with a 60× or 100× objective. This low DAPI concentration was used so as not to oversaturate the signal. Although DAPI shows that nuclear size and staining intensity are proportional to DNA content, the nuclear GFP signal is not proportional to either nuclear size or DNA content. *z*-stacks that covered the entire nucleus were acquired. All images were deconvolved using NIS-Elements software (Nikon). An area was drawn around the diploid or polyploid nucleus of selected in-focus stacks using the NIS-Elements draw bezier ROI tool, and the adaptive background compensation function was used to decrease background signal until the DAPI pixel density measurement was between 0 and 100 around each nucleus. The area quantified was defined by the DAPI signal, as the nuclear-localized GFP signal was found to be sometimes broader than the DAPI-stained DNA. DNA content was quantified for each nucleus by obtaining the sum intensity of the DAPI pixel density in NIS-Elements. For multinucleate SPG, the ploidy of each nucleus was measured, and the total cell ploidy was determined by summing the ploidy values for each nucleus within the cell. Ploidy was calculated by normalizing each SPG nucleus from wandering third instar larvae to diploid neuronal nuclei (average of 8-12 neurons per SPG cell). The diploid neurons were identified by staining with anti-ELAV (Robinow and White, 1991). For salivary glands, ploidy was calculated by normalizing each salivary gland nucleus from wandering third instar larvae (average of six nuclei per salivary gland) to the diploid imaginal ring cells (average of eight imaginal ring cells per gland). In both cases, diploid reference cells and polyploid cells were collected from the same animal and imaged on the same slide with the same exposure settings.

Statistical significance was determined by the Kruskal–Wallis test with Dunn's multiple comparisons or the Mann–Whitney two-tailed test using GraphPad Prism. Where necessary, the Kruskal–Wallis test was followed by a Bonferroni adjustment.

### Dye penetration assay and septate junction analysis

The Rhodamine-dextran dye penetration assay and septate junction analyses were performed in wandering third instar larvae from all genotypes listed in Table 2 and Fig. S6M. The dye penetration assay was carried out as described (Unhavaithaya and Orr-Weaver, 2012) with some modifications. Third instar larvae were placed on molasses agar plates and, immediately prior to dye injection, gently rolled between Kimwipe sheets. The larvae were transferred to double-sided tape, which was placed on microscope slides, and gently pressed down to stick the larvae to the tape. The slide was placed on an inverted microscope and larvae were injected in the posterior body cavity with a machine-drawn capillary needle filled with tetramethylrhodamine dextran (ThermoFisher Scientific, D1816, 10 kDa; 2.5 mM final concentration in water). Larvae were injected until the entire body turned the color of the dye. Injected larvae were covered with halocarbon oil for recovery and gently transferred to molasses plates after wiping off excess oil. After 10-15 min post injection, brains from live, injected larvae were dissected in unsupplemented Grace's medium, washed three times in the medium and mounted in Grace's medium along with some larval carcasses onto a microscope slide and coverslip. Brains were live imaged on a Nikon Eclipse inverted microscope using a 10× objective. A brain was called positive for dye penetration when there was detectable signal inside of the brain lobes that was clearly higher than the background signal. In cases in which dye clearly penetrated the brain, images of single frames in which the inside of the brain lobes were in focus were taken. In brains in which dye penetration was not as clear, *z*-stacks of the brain were taken and several focal planes were analyzed. Brains in which dye penetration was ambiguous were not used in the quantification. Three independent researchers examined and scored the brains. Visible NRXIV-GFP and/or DLG breaks were scored using *z*-stacks of deconvolved images taken with the Nikon Eclipse. Chi-square tests were used to evaluate whether the differences in dye penetration or in visible septate junctions breaks were significant between each of the control and experimental genotypes.

### Fluorescence *in situ* hybridization (FISH) or alkaline phosphatase (AP) *in situ* hybridization

Salivary glands were dissected from third instar larvae (96-120 h AED) and fixed, prehybridized, hybridized, washed, and detected as described (Wolff, 2000). Larval brains were dissected from third instar larvae (∼120 h AED) and processed for FISH as described (Lecuyer et al., 2008). Sense and antisense probes were generated by amplifying the corresponding cDNAs for all genes assayed from *Drosophila* gene collection clones (*Myc*, LD32539; *eIF4E*, SD05406; *Su(H)*, GH10914; *N*, LD34134; *stg*, LD47579) following the Berkeley Drosophila Genome Project protocol. Amplified PCR fragments were purified and *in vitro* transcribed. All probes were size-reduced with 2× carbonate buffer (120 mM Na_2_CO_3_, 80 mM NaHCO_3_, pH 10.2) for 15 min. AP detection time for *Myc* and *eIF4E* probes was 23 min. Both control *fkh-GAL4* alone and *Su(H)* RNAi salivary glands, distinguishable by size, were hybridized and histochemically labeled in the same dish. Thus, the intensity of the color stain can be compared between the two samples.

Intensity measurements of protein fluorescence, AP or FISH were performed with ImageJ. Further details are provided in the supplementary Materials and Methods.

### Cell area

SPG cell area was measured (μm^2^) by tracing the boundary of the cell, marked by NRXIV-GFP, using the NIS-Elements area tool. To determine statistical significance, Spearman's correlation was performed with linear regression.

### Statistics

Sample sizes were calculated using the pwr.t.test (for two groups) and pwr.anova.test (for more than two groups) functions in the ‘pwr’ R package, setting the power to 0.8 and the significance level to 0.05. Expected effect sizes were calculated from Cohen's d [using cohen.d() in the ‘effsize’ R package] with preliminary data, and sample sizes were increased by 20% to account for the use of non-parametric tests. All data observations are independent and meet the assumptions of the (non-parametric) statistical tests.

## Supplementary Material

Supplementary information

Supplementary information

## References

[DEV157115C1] Bailey A. P., Koster G., Guillermier C., Hirst E. M. A., MacRae J. I., Lechene C. P., Postle A. D. and Gould A. P. (2015). Antioxidant role for lipid droplets in a stem cell niche of Drosophila. *Cell* 163, 340-353. 10.1016/j.cell.2015.09.020 26451484 PMC4601084

[DEV157115C2] Bainton R. J., Tsai L. T., Schwabe T., DeSalvo M., Gaul U. and Heberlein U. (2005). *moody* encodes two GPCRs that regulate cocaine behaviors and blood-brain barrier permeability in Drosophila. *Cell* 123, 145-156. 16213219 10.1016/j.cell.2005.07.029

[DEV157115C3] Banerjee S., Blauth K., Peters K., Rogers S. L., Fanning A. S. and Bhat M. A. (2010). Drosophila Neurexin IV interacts with Roundabout and is required for repulsive midline axon guidance. *J. Neurosci.* 30, 5653-5667. 10.1523/JNEUROSCI.6187-09.2010 20410118 PMC2869042

[DEV157115C4] Bray S. J. (2006). Notch signalling: a simple pathway becomes complex. *Nat. Rev. Mol. Cell Biol.* 7, 678-689. 10.1038/nrm2009 16921404

[DEV157115C5] Contrino S., Smith R. N., Butano D., Carr A., Hu F., Lyne R., Rutherford K., Kalderimis A., Sullivan J., Carbon S. et al. (2012). modMine: flexible access to modENCODE data. *Nucleic Acids Res.* 40, D1082-D1088. 10.1093/nar/gkr921 22080565 PMC3245176

[DEV157115C6] Cross J. C. (2005). How to make a placenta: mechanisms of trophoblast cell differentiation in mice--a review. *Placenta* 26 Suppl. A, S3-S9. 15837063 10.1016/j.placenta.2005.01.015

[DEV157115C7] Deng W. M., Althauser C. and Ruohola-Baker H. (2001). Notch-Delta signaling induces a transition from mitotic cell cycle to endocycle in Drosophila follicle cells. *Development* 128, 4737-4746. 11731454 10.1242/dev.128.23.4737

[DEV157115C8] Edgar B. A. and O'Farrell P. H. (1990). The three postblastoderm cell cycles of Drosophila embryogenesis are regulated in G2 by string. *Cell* 62, 469-480. 10.1016/0092-8674(90)90012-4 2199063 PMC2753418

[DEV157115C9] Edgar B. A. and Orr-Weaver T. L. (2001). Endoreplication cell cycles: more for less. *Cell* 105, 297-306. 10.1016/S0092-8674(01)00334-8 11348589

[DEV157115C10] Edgar B. A., Zielke N. and Gutierrez C. (2014). Endocycles: a recurrent evolutionary innovation for post-mitotic cell growth. *Nat. Rev. Mol. Cell Biol.* 15, 197-210. 10.1038/nrm3756 24556841

[DEV157115C11] Fox D. T. and Duronio R. J. (2013). Endoreplication and polyploidy: insights into development and disease. *Development* 140, 3-12. 10.1242/dev.080531 23222436 PMC3513989

[DEV157115C12] Fox D. T., Gall J. G. and Spradling A. C. (2010). Error-prone polyploid mitosis during normal Drosophila development. *Genes Dev.* 24, 2294-2302. 10.1101/gad.1952710 20952538 PMC2956208

[DEV157115C13] Frawley L. E. and Orr-Weaver T. L. (2015). Polyploidy. *Curr. Biol.* 25, R353-R358. 10.1016/j.cub.2015.03.037 25942544

[DEV157115C14] Hamada S. and Fujita S. (1983). DAPI staining improved for quantitative cytofluorometry. *Histochemistry* 79, 219-226. 10.1007/BF00489783 6643138

[DEV157115C15] Henderson K. D. and Andrew D. J. (2000). Regulation and function of *Scr*, *exd*, and *hth* in the Drosophila salivary gland. *Dev. Biol.* 217, 362-374. 10.1006/dbio.1999.9560 10625560

[DEV157115C16] Housden B. E., Fu A. Q., Krejci A., Bernard F., Fischer B., Tavaré S., Russell S. and Bray S. J. (2013). Transcriptional dynamics elicited by a short pulse of Notch activation involves feed-forward regulation by *E(spl)/Hes* genes. *PLoS Genet.* 9, e1003162 10.1371/journal.pgen.1003162 23300480 PMC3536677

[DEV157115C17] Lecuyer E., Necakov A. S., Caceres L. and Krause H. M. (2008). High-resolution fluorescent in situ hybridization of Drosophila embryos and tissues. *CSH Protoc*. 2008, pdb prot5019. 21356853 10.1101/pdb.prot5019

[DEV157115C18] Lee Y. U., Son M., Kim J., Shim Y. H. and Kawasaki I. (2016). CDC-25.2, a *C. elegans* ortholog of cdc25, is essential for the progression of intestinal divisions. *Cell Cycle* 15, 654-666. 27104746 10.1080/15384101.2016.1146839 PMC4845952

[DEV157115C19] Li D., Liu Y., Pei C., Zhang P., Pan L., Xiao J., Meng S., Yuan Z. and Bi X. (2017). miR-285-Yki/Mask double-negative feedback loop mediates blood-brain barrier integrity in Drosophila. *Proc. Natl. Acad. Sci. USA* 114, E2365-E2374. 10.1073/pnas.1613233114 28265104 PMC5373330

[DEV157115C20] Limmer S., Weiler A., Volkenhoff A., Babatz F. and Klämbt C. (2014). The Drosophila blood-brain barrier: development and function of a glial endothelium. *Front. Neurosci.* 8, 365 10.3389/fnins.2014.00365 25452710 PMC4231875

[DEV157115C21] Lopez-Schier H. and St Johnston D. (2001). Delta signaling from the germ line controls the proliferation and differentiation of the somatic follicle cells during Drosophila oogenesis. *Genes Dev.* 15, 1393-1405. 10.1101/gad.200901 11390359 PMC312703

[DEV157115C22] Micchelli C. A. and Perrimon N. (2006). Evidence that stem cells reside in the adult Drosophila midgut epithelium. *Nature* 439, 475-479. 10.1038/nature04371 16340959

[DEV157115C23] Myer D. L., Bahassi el M. and Stambrook P. J. (2005). The Plk3-Cdc25 circuit. *Oncogene* 24, 299-305. 10.1038/sj.onc.1208278 15640846

[DEV157115C24] Nagl W. (1978). *Endopolyploidy and Polyteny in Differentiation and Evolution: Towards an Understanding of Quantitative Variation of Nuclear DNA in Ontogeny and Phylogeny*. Amsterdam, The Netherlands: North-Holland Publishing Company.

[DEV157115C25] Ohlstein B. and Spradling A. (2006). The adult Drosophila posterior midgut is maintained by pluripotent stem cells. *Nature* 439, 470-474. 10.1038/nature04333 16340960

[DEV157115C26] Orr-Weaver T. L. (2015). When bigger is better: the role of polyploidy in organogenesis. *Trends Genet.* 31, 307-315. 10.1016/j.tig.2015.03.011 25921783 PMC4537166

[DEV157115C27] Pandit S. K., Westendorp B., Nantasanti S., van Liere E., Tooten P. C. J., Cornelissen P. W. A., Toussaint M. J. M., Lamers W. H. and de Bruin A. (2012). E2F8 is essential for polyploidization in mammalian cells. *Nat. Cell Biol.* 14, 1181-1191. 10.1038/ncb2585 23064264

[DEV157115C28] Ravid K., Lu J., Zimmet J. M. and Jones M. R. (2002). Roads to polyploidy: the megakaryocyte example. *J. Cell Physiol.* 190, 7-20. 10.1002/jcp.10035 11807806

[DEV157115C29] Robinow S. and White K. (1991). Characterization and spatial distribution of the ELAV protein during *Drosophila melanogaster* development. *J. Neurobiol.* 22, 443-461. 10.1002/neu.480220503 1716300

[DEV157115C30] Rossant J. and Cross J. C. (2001). Placental development: lessons from mouse mutants. *Nat. Rev. Genet.* 2, 538-548. 10.1038/35080570 11433360

[DEV157115C31] Salle J., Campbell S. D., Gho M. and Audibert A. (2012). CycA is involved in the control of endoreplication dynamics in the Drosophila bristle lineage. *Development* 139, 547-557. 10.1242/dev.069823 22223681

[DEV157115C32] Schoenfelder K. P., Montague R. A., Paramore S. V., Lennox A. L., Mahowald A. P. and Fox D. T. (2014). Indispensable pre-mitotic endocycles promote aneuploidy in the Drosophila rectum. *Development* 141, 3551-3560. 10.1242/dev.109850 25142462 PMC6517832

[DEV157115C33] Schulte J., Tepass U. and Auld V. J. (2003). Gliotactin, a novel marker of tricellular junctions, is necessary for septate junction development in Drosophila. *J. Cell Biol.* 161, 991-1000. 10.1083/jcb.200303192 12782681 PMC2172969

[DEV157115C34] Schwabe T., Bainton R. J., Fetter R. D., Heberlein U. and Gaul U. (2005). GPCR signaling is required for blood-brain barrier formation in Drosophila. *Cell* 123, 133-144. 10.1016/j.cell.2005.08.037 16213218

[DEV157115C35] Skalska L., Stojnic R., Li J., Fischer B., Cerda-Moya G., Sakai H., Tajbakhsh S., Russell S., Adryan B. and Bray S. J. (2015). Chromatin signatures at Notch-regulated enhancers reveal large-scale changes in H3K56ac upon activation. *EMBO J.* 34, 1889-1904. 10.15252/embj.201489923 26069324 PMC4547894

[DEV157115C36] Slattery M., Ma L., Spokony R. F., Arthur R. K., Kheradpour P., Kundaje A., Negre N., Crofts A., Ptashkin R., Zieba J. et al. (2014). Diverse patterns of genomic targeting by transcriptional regulators in *Drosophila melanogaster*. *Genome Res.* 24, 1224-1235. 10.1101/gr.168807.113 24985916 PMC4079976

[DEV157115C37] Song Y. and Lu B. (2011). Regulation of cell growth by Notch signaling and its differential requirement in normal vs. tumor-forming stem cells in Drosophila. *Genes Dev.* 25, 2644-2658. 22190460 10.1101/gad.171959.111 PMC3248685

[DEV157115C38] Spéder P. and Brand A. H. (2014). Gap junction proteins in the blood-brain barrier control nutrient-dependent reactivation of Drosophila neural stem cells. *Dev. Cell* 30, 309-321. 10.1016/j.devcel.2014.05.021 25065772 PMC4139190

[DEV157115C39] Spradling A. C. (1993). Developmental genetics of oogenesis. In *The Development of Drosophila Melanogaster* (ed. Bate M. and Martinez Arias A.), pp. 1-70. Cold Spring Harbor, NY: Cold Spring Harbor Laboratory Press.

[DEV157115C40] Stork T., Engelen D., Krudewig A., Silies M., Bainton R. J. and Klämbt C. (2008). Organization and function of the blood-brain barrier in Drosophila. *J. Neurosci.* 28, 587-597. 10.1523/JNEUROSCI.4367-07.2008 18199760 PMC6670337

[DEV157115C41] Taniguchi K., Kokuryo A., Imano T., Minami R., Nakagoshi H. and Adachi-Yamada T. (2012). Binucleation of Drosophila adult male accessory gland cells increases plasticity of organ size for effective reproduction. *Biol. Syst. Open Access* 1, 1-5. 10.4172/2329-6577.1000e101

[DEV157115C42] Terriente-Felix A., Li J., Collins S., Mulligan A., Reekie I., Bernard F., Krejci A. and Bray S. (2013). Notch cooperates with Lozenge/Runx to lock haemocytes into a differentiation programme. *Development* 140, 926-937. 10.1242/dev.086785 23325760 PMC3557782

[DEV157115C43] Trakala M., Rodríguez-Acebes S., Maroto M., Symonds C. E., Santamaría D., Ortega S., Barbacid M., Méndez J. and Malumbres M. (2015). Functional reprogramming of polyploidization in megakaryocytes. *Dev. Cell* 32, 155-167. 10.1016/j.devcel.2014.12.015 25625205

[DEV157115C44] Unhavaithaya Y. and Orr-Weaver T. L. (2012). Polyploidization of glia in neural development links tissue growth to blood-brain barrier integrity. *Genes Dev.* 26, 31-36. 10.1101/gad.177436.111 22215808 PMC3258963

[DEV157115C45] Volkenhoff A., Weiler A., Letzel M., Stehling M., Klämbt C. and Schirmeier S. (2015). Glial glycolysis is essential for neuronal survival in Drosophila. *Cell Metab.* 22, 437-447. 10.1016/j.cmet.2015.07.006 26235423

[DEV157115C46] Watson E. D. and Cross J. C. (2005). Development of structures and transport functions in the mouse placenta. *Physiology* 20, 180-193. 10.1152/physiol.00001.2005 15888575

[DEV157115C47] Wolff T. (2000). Histological techniques for the Drosophila eye part I: larva and pupae. In *Drosophila Protocols* (ed. Sullivan W., Ashburner M. and Hawley R. S.), pp. 216-220. Cold Spring Harbor, NY: Cold Spring Harbor Laboratory Press.

