# Supplementary material for: Variant cell cycles regulated by Notch signaling control cell size and ensure a functional blood-brain barrier
Source: Development. 2018 Feb 1;145(3):dev157115. doi: 10.1242/dev.157115 (PMC5818001; doi:10.1242/dev.157115)
Supplement: Supplementary information [file develop-145-157115-s1.pdf]

SUPPLEMENTAL MATERIALS AND METHODS, TABLES and FIGURES

SUPPLEMENTAL MATERIALS AND METHODS

Intensity measurements of protein fluorescence, alkaline phosphatase (AP) or fluorescence in situ hybridization (FISH)

Anti-Cyclin B cytoplasmic fluorescence in both first and second instar larval brain SPG and salivary glands, *Myc* and *eIF4E* AP *in situ* signals in salivary glands or *N*, *Su(H)* and *stg in situ* hybridization fluorescence in brain lobes were all measured with Image J. For brain lobe or salivary gland protein or FISH transcript measurements, a single frame from Z-stacks taken with an LSM710 confocal microscope containing the SPG or the salivary gland nucleus in focus was used. For AP *in situ* measurements, a single scan of salivary glands taken with a Zeiss SteREO Discovery V8 microscope was used. For SPG, measurements were made from the signal from the entire circumference of whole half brain lobes. For salivary glands, measurements were made from the entire area from half salivary glands. All samples were imaged using the same exposure times between first and second instar stages (anti-Cyclin B fluorescence) or between the control and RNAi samples (AP signal and FISH fluorescence). Three independent background measurements were taken in areas without fluorescence or AP signal. Image J "area", "integrated density", and "mean gray value measurements" settings were selected for all measurements. Values were obtained from the following formula: *Corrected total fluorescence or AP signal= integrated density- (area of selected cell X mean fluorescence or AP signal of background readings)*. To determine the statistical significance between control and RNAi samples the Mann-Whitney, two-tailed test was applied using GraphPad Prism.

EdU and Phospho-Histone H3 (PHH3) labeling

EdU labeling was performed in salivary glands from 96-120 hrs after egg deposition (AED) third instar larva or in brains from mid-late (30-40 hrs AED) first instar larvae. All tissues were dissected in unsupplemented Grace's medium and

Development • Supplementary information

incubated in 50µM EdU in Grace's medium for 1 hour at room temperature. EdU and PHH3 samples were fixed in 1X PBS/4% paraformaldehyde for 30 minutes at room temperature. After fixation, samples were washed 4X for 10 min with 1X PBT (PBS + 0.3% TritonX-100). Larval brains were then blocked in 1X blocking solution (1X PBST-0.3%, 5% goat and donkey serum) for 1 hr, incubated with GFP-Booster nanobody [1:400] or anti-PHH3 [1:400] in blocking solution overnight at 4°C and washed 4X for 20 min with 1X PBT. Right before detection, both larval salivary glands and brain samples were rinsed 2X in 1X PBS, and incubated in Alexa Fluor 594 or 555 Click-it EdU (Invitrogen) reaction cocktail following the manufacture's instructions or with Alexa-647 anti-rabbit for 1 hour at room temperature in blocking solution. Samples were washed extensively with 1X PBT (6X for 20 min) and stained with DAPI [50 ng/ml] for 15 min at room temperature, washed with 1X PBT, and mounted in Vectashield (Vector laboratories).

Salivary glands chromosome squashes

Polytene chromosomes squashes were prepared following the protocol in (Cai et al. 2010). Squashed chromosomes were stained with DAPI [50 ng/ml].

SUPPLEMENTAL REFERENCE

Cai W, Jin Y, Girton J, Johansen J, Johansen KM. 2010. Preparation of Drosophila polytene chromosome squashes for antibody labeling. *J. Vis. Exp*: 1-4.

Development • Supplementary information

Supplemental Table S1: Abbreviation and full genotypes used throughout the manuscript

Abbreviation	Full genotype
GAL4 control (brain)	*+; moody-GAL4, UAS-GFP^{nls}/+; NRXIV::GFP/+*
Dcr2 OE (brain)	*UAS-Dicer2/+; moody-GAL4, UAS-GFP^{nls}/+; NRXIV::GFP/+*
Su(H) RNAi (brain)	*UAS-Suppressor of Hairless* RNAi*/+; moody-GAL4, UAS-GFP^{nls}/+; NRXIV::GFP/+*
N RNAi (brain)	*+; moody-GAL4, UAS-GFP^{nls}/UAS-Notch* RNAi*; NRXIV::GFP/+*
Dcr2 OE; *Dl* RNAi (brain)	*UAS-Dicer2/+; moody-GAL4, UAS-GFP^{nls}/UAS-Delta* RNAi*; NRXIV::GFP/+*
Dcr2 OE; *stg* RNAi (brain)	*UAS-Dicer2/+; moody-GAL4, UAS-GFP^{nls}/+; NRXIV::GFP/UAS-string RNAi*
stg OE (brain)	*+; moody-GAL4, UAS-GFP^{nls}/+; NRXIV::GFP/UAS-string*
cycB RNAi (brain)	*+; moody-GAL4, UAS-GFP^{nls}/+; NRXIV::GFP/UAS-cyclinB* RNAi
fkh-GAL4 control (SG)	*+; +; forkhead-GAL4/+*
Su(H) RNAi (SG)	*UAS-Suppressor of Hairless* RNAi*/+; +; forkhead-GAL4/+*
Myc rescue (SG)	*UAS-Suppressor of Hairless* RNAi*/+; UAS-Myc/+; forkhead-GAL4/+*

Development • Supplementary information

Supplemental Table S2: SPG nuclear number, cell area and ploidy (20C-32C range)

	Nuclei number	Cell area (μm^2)	Ploidy (C value)
SPG 1	1	7800	23
SPG 2	1	6700	24
SPG 3	1	7200	25
SPG 4	1	5800	22
SPG 5	1	9000	22
SPG 6	1	9400	24
SPG 7	1	6100	21
SPG 8	1	7400	22
SPG 9	1	6300	20
SPG 10	1	6500	22
SPG 11	2	15000	28
SPG 12	2	7700	21
SPG 13	2	5800	31
SPG 14	2	8000	24
SPG 15	2	6200	21
SPG 16	2	17000	24
SPG 17	2	7200	22
SPG 18	2	7600	29
SPG 19	2	7500	27
SPG 20	2	12000	25
SPG 21	4	13000	29
SPG 22	4	8800	29
SPG 23	4	11000	26
SPG 24	4	8500	31
SPG 25	4	8000	30
SPG 26	4	12000	25
SPG 27	4	9900	26
SPG 28	4	11000	27
SPG 29	4	9300	24
SPG 30	4	11000	29
SPG 31	4	12000	30

Development • Supplementary information

SUPPLEMENTAL FIGURES

Von Stetina_FigS1

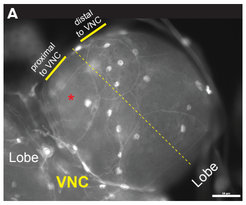

B Quantification of Proximity of Mononucleate SPG to VNC

Samples scored	Percentage of lobes with mononucleate SPG proximal to VNC	Percentage of mononucleate SPG proximal to VNC
28 brains	**97%** (33/34 lobes)	**97%** (62/64 SPG cells)

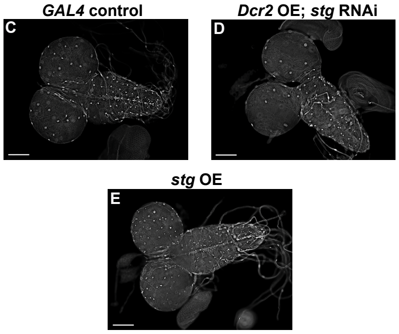

Supplemental Figure S1. Analysis of the position of mononucleate SPG relative to the VNC. (A) Brain lobes from wandering third instar larvae were analyzed for the number of mononucleate SPG cells adjacent to or distal to the VNC. This was done by drawing a line on the middle of each brain hemisphere image and scoring the number of SPG on the side adjacent to the VNC versus distal. Images were acquired from either dorsal or ventral sides of the lobes, depending on where mononucleate SPG cells were found. In 97% percent of all the lobes examined (B), mononucleate cells were found proximal to the VNC rather than distal (n=33 lobes, two biological replicates) (Chi-squared test, $P= 4.1\times10^{-8}$). Red asterisk marks the mononucleate SPG found in the half lobe shown. Scale bar, 50μm. (C-E) Whole brains from *GAL4* control (C), *Dcr2* OE; *stg* RNAi (D), and *stg* overexpression (OE). Scale bars, 100μm.

Development • Supplementary information

Von Stetina_FigS2

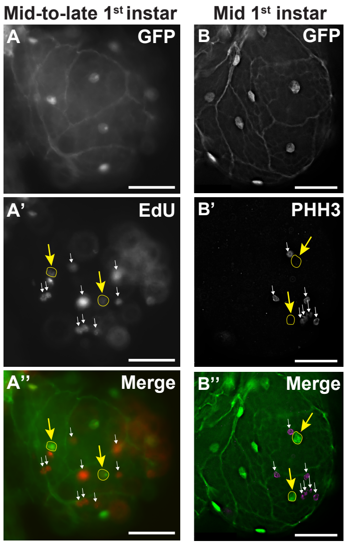

Supplemental Figure S2. DNA replication but no mitotic markers are detected in first instar SPG prior to the switch to endomitosis. (A-A'') Brain lobe from mid-to-late first instar larvae (~30-40 hrs AED) stained with anti-GFP (A; marks both nuclei and cell boundaries) and labeled with EdU (A') to mark SPG in S phase. Nuclei also were identified by DAPI staining (not shown). (A'') GFP (green) and EdU (red) merged channels. We detected EdU incorporation in 12% of the SPG (n=39 brains, 459 SPG, one biological replicate). Yellow arrows point to two SPG nuclei positive for EdU (circled). The white arrows show EdU-labeled nuclei in other tissue layers in the brain. (B-B'') Brain lobe from mid-first instar larvae (~36 hrs AED) stained with anti-GFP (B) and phosphorylated-histone H3 (PHH3) (B'). (B'') GFP (green) and PHH3 (magenta) merged channels. No PHH3 positive SPG were detected at this developmental time point (n=19 brains, 257 SPG, one biological replicate). Yellow arrows and circles point to PHH3 positive SPG nuclei in the lobe. White arrows show mitotic cells in other tissue layers in the brain. Scale bars in all panels, 25 μm.

Von Stetina_FigS3

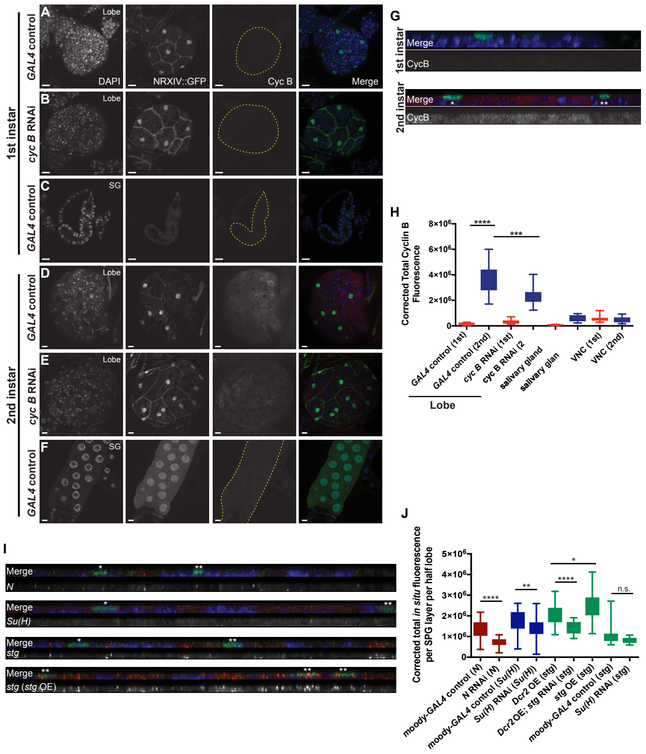

Supplemental Figure S3. Cyclin B protein levels are upregulated in SPG in brain lobes at the second instar larval stage. (A-F) Single confocal scans of larval brain lobes or salivary glands stained with DAPI (first column, blue in merge), anti-GFP (second column; marks both SPG or salivary gland nuclei and SPG cell boundaries, green in merge) and anti-Cyclin B (CycB) (third column, red in merge). (A, C) First instar larval brain lobe from *GAL4* control showing basal CycB protein levels in SPG and in polyploid salivary gland cells from same developmental stage. (D) Larval brain lobe from *GAL4* control second instar larva showing increased CycB signal in SPG at the developmental window when the transition to multiple nuclei occurs. (F) CycB levels in second instar larval salivary gland. (B, E) Larval brain lobe from first instar *cycB* RNAi larvae showing basal CycB levels at the first instar stage but significantly decreased levels of CycB at the second instar larval stage. Dotted lines in third columns of panels A-C and F outline brain lobes and salivary glands. Scale bars, 10 µm. (G) Orthogonal views of first and second instar larval brains showing CycB protein levels in the SPG layer (bottom panels). Top panels show the merge with nuclear localized GFP (green), anti-CycB (red) and DAPI (blue). (H) Graph of corrected total anti-CycB fluorescence from single scanned images for each indicated genotype and sample. First instar *GAL4* control larval brain lobes (n=15 brains), salivary glands (n=5), VNCs (n=18), *cycB* RNAi larval brain lobes (n=13 brains); second instar *GAL4* control larval brain lobes (n=12 brains), salivary glands (n=5), VNCs (n=19), *cycB* RNAi larval brain lobes (n=17 brains). Data from one biological replicate. Mann-Whitney, two-tailed test, ***P=0.0003; ****P<0.0001. Although the *cycB* RNAi establishes the specificity of the CycB antibody staining, it did not eliminate protein and did not affect endomitosis. (I-J) Fluorescence *in situ* hybridization (FISH) analysis to determine the efficiency of RNAi knockdown in SPG. (I) Orthogonal views of third instar (~120 hrs AED) brain lobe FISH samples showing *N*, *Su(H)* or *stg* transcripts (bottom panels) in the SPG layer. Top panels show the merge with nuclear localized GFP (green), FISH signal (red) and DAPI (blue). (J) Graph of corrected total *in situ* fluorescence from single scanned images for each individual genotype and sample from third instar larva. *GAL4* control (*N* probe: n=20 half lobes, 20 brains; *Su(H)*

Development • Supplementary information

probe: n=33 half lobes, 19 brains; *stg* probe: n=18 half lobes, 17 brains); *N* RNAi (*N* probe: n= 22 half lobes, 22 brains); *Su(H)* RNAi (*Su(H)* probe: n= 43 half lobes, 29 brains; *stg* probe: n=18 half lobes, 16 brains); *stg* OE (*stg* probe: n= 32 half lobes, 14 brains); *Dcr2* OE (*stg* probe: n= 32 half lobes, 21 brains); *Dcr2* OE; *stg* RNAi (*stg* probe: n=25 half lobes, 19 brains). Y axis, *in situ* fluorescence arbitrary units. Data from one biological replicate. Mann-Whitney, two-tailed test, *P=0.0333; **P<0.0030; ****P<0.0001; n.s.=not significant. In both G and I, asterisks mark GFP-labeled nuclei from mononucleate SPG (*) or from multinucleate SPG (**). Complete genotypes are in Supplemental Table S1.

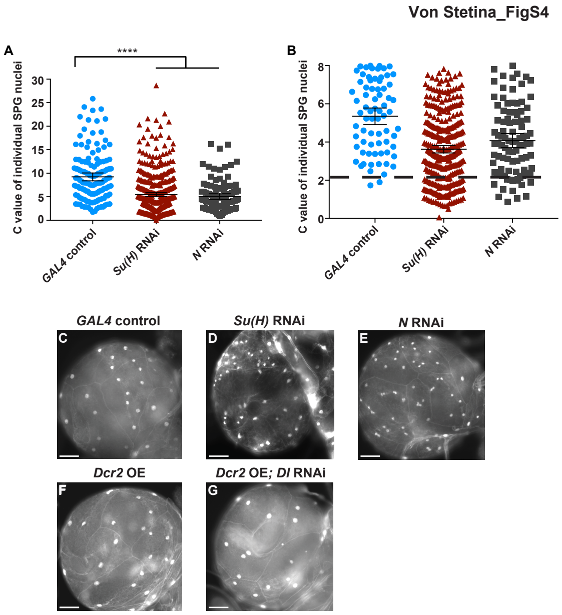

Supplemental Figure S4. Decreased Notch signaling results in a larger population of SPG cells having a C value equal to or less than diploid 2C. (A) Scatter plot showing the DNA ploidy C values of individual nuclei from multinucleate (endomitotic) SPG in brain lobes of wandering third instar larvae. *GAL4* control, n=147 nuclei, 44 SPG, 33 brains; *Su(H)* RNAi, n=436 nuclei, 44 SPG, 25 brains; *N* RNAi, n=110 nuclei, 17 SPG, 17 brains. Data from three biological replicates. Kruskal-Wallis with Dunn's multiple comparisons test, **** $P < 0.0001$, mean ± 95% c.i.. (B) Scatter plot showing the DNA ploidy C values of individual endomitotic nuclei in the brain lobe with equal to or less than 8C values. The data are a subset of the data from panel A with the axes expanded so that nuclei with less than 2C ploidy can be easily visualized. The percentage of nuclei with less than 2C were calculated from the data in panel A, not the subset of data in panel B. Dotted line indicates the diploid 2C value. (C-G) Brain lobes from wandering third instar larvae in which Notch signaling was perturbed. *GAL4* control (C), *Su(H)* RNAi (D), *N* RNAi (E), *Dcr2* OE (F), and *Dcr2* OE; *Dl* RNAi (G). Scale bars, 100 μm. See Supplemental Table S1 for complete genotypes.

Development • Supplementary information

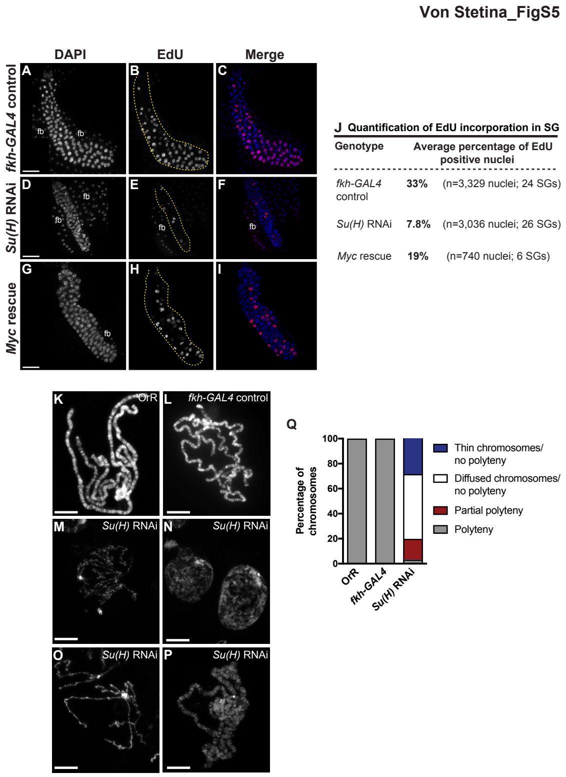

Supplemental Figure S5. ***Su(H)*** **RNAi salivary glands have S-phase and chromosomal defects.** (A-J) *Su(H)* RNAi salivary glands have a reduction of nuclei in S-phase. *fkh-GAL4* alone control (A-C), *Su(H)* RNAi (D-F) or *Su(H)* RNAi*; UAS-Myc* rescued (G-I) salivary glands from 96-120 hours AED third instar larvae were labeled with EdU (Alexa-594, red) to monitor nuclei in S phase and with DAPI to stain DNA (blue). Panels A, D, G DAPI; panels B, E, H EdU; panels C, F, I DAPI and EdU merged. Yellow dotted lines outline the salivary glands in panels B, E and H. fb, fat body. Scale bars, 100 μm. (J) Quantification of EdU incorporation in salivary glands for control, *Su(H)* RNAi and *Myc* rescue from one biological replicate. (K-Q) *Su(H)* RNAi chromosomes have morphological defects. Images of salivary gland chromosome squashes from wandering third instar larvae stained with DAPI (shown in white). OrR (K) and *fkh-GAL4* alone (L) control chromosome spreads illustrating normal thickness and the banding pattern representative of polyteny. (M-P) *Su(H)* RNAi chromosome spreads showing different defects including thinning (M) or diffusion (N) of the chromosomes, or partial polyteny (O). A small percentage of the *Su(H)* RNAi chromosomes appear relatively normal (P). Scale bars, 20 μm. (Q) Quantification of polytene chromosome morphology. (*OrR* control, n=600 chromosomes; *fkh-GAL4* alone control, n=600 chromosomes; *Su(H)* RNAi, n=629 chromosomes, one biological replicate). See Supplemental Table S1 for complete genotypes.

Von Stetina_FigS6

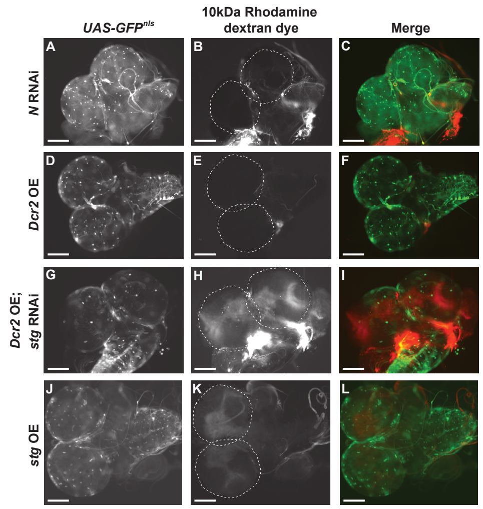

M Quantification of Visible Breaks in Septate Junction Markers

Genotype	NRXIV breaks	DLG breaks	Both NRXIV and DLG breaks	N
GAL4 control	0%	7%	0%	30
Su(H) RNAi	19%	35%	19%	31
stg OE	10%	23%	6%	31
Dcr2 OE	0%	10%	0%	29
Dcr2 OE; *stg* RNAi	14%	31%	14%	29

Supplemental Figure S6. Dye penetration of the BBB and visible breaks in the septate junctions are observed when the percentage of endomitotic SPG is altered. Dye penetration assays were performed in brains of wandering third instar larvae in which different transgenes were driven in the SPG by *moody-GAL4*. (A-C) In 71% of animals no dye signal was detected inside the lobes expressing *N* RNAi. (D-F) In 80% of animals no dye signal was detected inside the *Dcr2* OE lobes. (G-I) In 52% of larvae dye penetrated the lobes expressing *Dcr2* OE; *stg* RNAi. (J-L) In 50% of animals dye penetrated the lobes with *stg* overexpression (OE). The dashed lines in the rhodamine dextran panels mark the edges of the brain lobes. For N and *P* values see Figure 5. Scale bars, 100 µm. (M) Quantification of visible breaks in the septate junction markers NRXIV and DLG for the genotypes listed from brain lobes of wandering third instar larvae. Relative to *GAL4* control, the visible breaks in DLG, NRXIV, and both DLG and NRXIV were significantly increased for *Su(H)* RNAi (Chi-squared test, *P*=0.0017, *P*=0.0033, and *P*=0.0033, respectively). No significant difference was found for *stg* OE brains (Chi-squared test, *P*=0.080, *P*=0.081, *P*=0.16). Relative to *Dcr2* OE, the visible breaks in NRXIV and both DLG and NRXIV were significantly increased for *Dcr2* OE; *stg* RNAi (Chi-squared test, *P*=0.038 and *P*=0.038, respectively; DLG, *P*=0.052). *GAL4* control and *Su(H)* RNAi data from four biological replicates; *stg* OE and *Dcr2* OE data from three biological replicates; *Dcr2* OE; *stg* RNAi from five biological replicates. Complete genotypes are in Supplemental Table S1

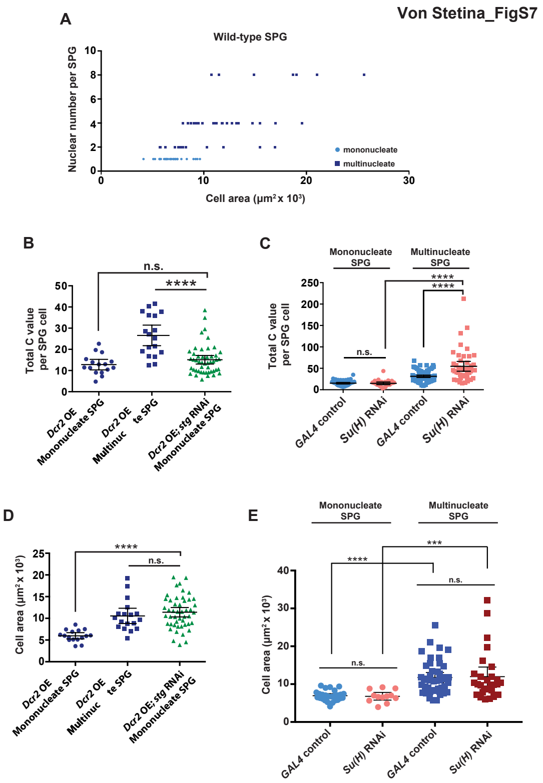

Development • Supplementary information

Supplemental Figure S7. Relationship between nuclear number, ploidy and cell area. All brains were collected from wandering third instar larvae. (A) Increased nuclear number in SPG correlates with increased cell area. Cell area (µm^2 x 10^3) in relation to SPG nuclear number (n=70 SPG; 36 brains, three biological replicates). Spearman correlation coefficient *r*=0.60, *P*<0.0001. (B) Scatter plot showing the DNA ploidy C values of individual endocycling and endomitotic SPG cells in *Dcr2* OE versus *Dcr2* OE; *stg* RNAi brain lobes. Mann-Whitney, two-tailed test, ****P < 0.0001; n.s.=not significant. *Dcr2* OE: n=16 endocycling SPG, 18 endomitotic SPG, 15 brains, one biological replicate. *Dcr2* OE; *stg* RNAi: n=48 endocycling SPG, 15 brains, one biological replicate. (C) Scatter plot showing the DNA ploidy C values of individual endocycling and endomitotic SPG cells in *GAL4* control versus *Su(H)* RNAi brain lobes. The control data are from Figure 6. *Su(H)* RNAi: n=33 endocycling SPG, 24 brains; n=44 endomitotic SPG, 33 brains, six biological replicates. Mann-Whitney, two-tailed test, ****P<0.0001; n.s.=not significant. (D) Scatter plot showing cell areas (µm^2 x 10^3) of individual endocycling and endomitotic SPG in *Dcr2* OE versus *Dcr2* OE; *stg* RNAi brain lobes. Mann-Whitney, two-tailed test, **** P< 0.0001; n.s.=not significant. *Dcr2* OE: n=16 endocycling SPG, 18 endomitotic SPG, 15 brains, one biological replicate. *Dcr2* OE; *stg* RNAi: n=48 endocycling SPG, 15 brains, one biological replicate. (E) Scatter plot showing cell area (µm^2 x 10^3) of individual *GAL4* control and *Su(H)* RNAi endocycling and endomitotic SPG cells in the brain lobe. Data from *GAL4* controls are the same presented in Figure 6D. *Su(H)* RNAi: n=12 endocycling SPG, 12 brains, n=28 endomitotic SPG, 15 brains, three biological replicates. Mann-Whitney, two-tailed test, ***P=0.0002; ****P<0.0001; n.s.=not significant. Scatter plots, mean ± 95% c.i.. See Supplemental Table S1 for complete genotypes.
